# Review of Ge(GeSn) and InGaAs Avalanche Diodes Operating in the SWIR Spectral Region

**DOI:** 10.3390/nano13030606

**Published:** 2023-02-02

**Authors:** Yuanhao Miao, Hongxiao Lin, Ben Li, Tianyu Dong, Chuangqi He, Junhao Du, Xuewei Zhao, Ziwei Zhou, Jiale Su, He Wang, Yan Dong, Bin Lu, Linpeng Dong, Henry H. Radamson

**Affiliations:** 1 Research and Development Center of Optoelectronic Hybrid IC, Guangdong Greater Bay Area Institute of Integrated Circuit and System, Guangzhou 510535, China; 2 Key Laboratory of Microelectronic Devices Integrated Technology, Institute of Microelectronics, Chinese Academy of Sciences, Beijing 100029, China; 3 Institute of Microelectronics, University of Chinese Academy of Sciences, Beijing 100049, China; 4 School of Physics and Information Engineering, Shanxi Normal University, Linfen 041004, China; 5 Shaanxi Province Key Laboratory of Thin Films Technology Optical Test, Xi’an Technological University, Xi’an 710032, China

**Keywords:** APDs, PDs, Ge(GeSn), InGaAs, group IV, LiDAR

## Abstract

Among photodetectors, avalanche photodiodes (APDs) have an important place due to their excellent sensitivity to light. APDs transform photons into electrons and then multiply the electrons, leading to an amplified photocurrent. APDs are promising for faint light detection owing to this outstanding advantage, which will boost LiDAR applications. Although Si APDs have already been commercialized, their spectral region is very limited in many applications. Therefore, it is urgently demanded that the spectral region APDs be extended to the short-wavelength infrared (SWIR) region, which means better atmospheric transmission, a lower solar radiation background, a higher laser eye safety threshold, etc. Up until now, both Ge (GeSn) and InGaAs were employed as the SWIR absorbers. The aim of this review article is to provide a full understanding of Ge(GeSn) and InGaAs for PDs, with a focus on APD operation in the SWIR spectral region, which can be integrated onto the Si platform and is potentially compatible with CMOS technology.

## 1. Introduction

**Figure 1:**
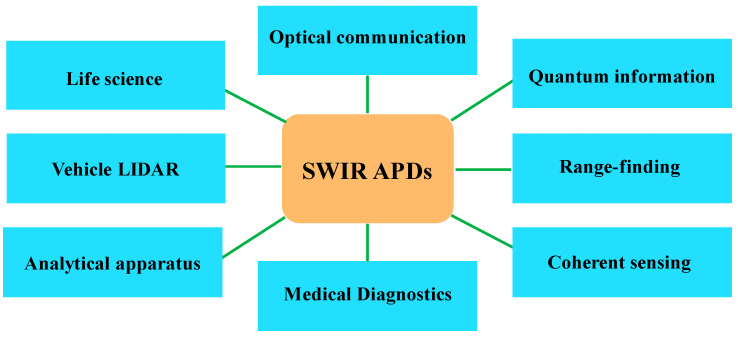
Potential applications of SWIR APDs in various fields.

Photodetectors convert the optical signal into an electrical signal, thereby amplifying the photocurrent through external or built-in gain processes. Generally, photodetector structures are classified as: photoconductors, PN photodiodes, PIN photodiodes, avalanche photodiodes (APDs), phototransistors, Schottky barrier photodiodes, metal-semiconductor-metal (MSM) photodiodes, etc. [1,2,3,4,5,6]. As a class of extremely sensitive semiconductor detectors, APDs are capable of multiplying the charge carriers via impact ionization, increasing the photocurrent that flows in response to a certain light power. The photocarriers generated by impact ionization can themselves initiate further impact ionization, resulting in the avalanche. When the reverse biased voltage is relatively small, APDs work in linear mode, and the electrical response is directly proportional to the incident light power, which makes linear mode APD is widely used in optical communication systems. However, increasing the reverse-biased voltage will push the APD into Geiger mode, for which the electrical response is non-linear. Thus, incident photons result in a large sudden voltage spike, indicating Geiger mode APDs as the most suitable for the photon counting system, and cryogenic cooling is unnecessary. Generally, APDs can be classified in terms of their operation wavelength range, compromising near-infrared (NIR, 0.78~1.1 μm), short-wavelength infrared (SWIR, 1.1–3 μm), mid-wavelength infrared (MWIR, 3–5 μm), and long-wavelength infrared (LWIR, 8–14 μm). Especially for the SWIR band, APDs have several potential applications, including optical communication, quantum information, range-finding, coherent sensing, medical diagnostics, analytical apparatus, vehicle light detection and ranging (LIDAR), life science, etc. (Figure 1) [7,8,9,10,11,12].

Among all the above-mentioned applications, vehicle LIDAR has the largest market share. With the rapid development of science and technology, vehicles have become an important part of daily life. In recent decades, consumers have been shifting their initial needs for basic vehicle functions towards high-level demands, such as safety and intelligence. Thus, an increasing number of vehicle manufacturers are looking for high-tech solutions to solve the safety and intelligence problems, aiming to make car safety systems more and more intelligent. To develop the advanced driving assistance system (ADAS) or automated driving system (ADS), advanced sensor technologies are needed. Up until now, there have been four types of detection techniques used in LIDAR systems, such as, direct detection (photodetector, linear-mode APD), photo counting (Geiger mode APD, SPAD), integrating direct (CMOS), and coherent detection. From a practical application perspective, sensors with a reasonable price and high reliability are important after entering the market. Herein, APDs are consequently the most mature technology.

**Figure 2:**
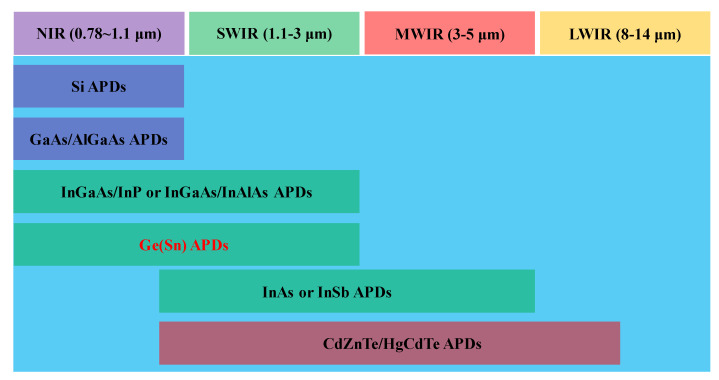
Typical semiconductor APDs versus infrared waveband.

Figure 2 shows the typical semiconductor APDs as a function of infrared waveband. Similar to other semiconductor devices, the first commercialized APDs were achieved from Si due to its long-term investment in Si process technology [13,14]. Both linear-mode and Geiger-mode (expressed as single photon avalanche diodes, SPADs) Si APDs feature extremely high performance, which ascribes to their ionization rate ratio, which varies considerably with electric fields. Although several pioneering studies on GaAs/AlGaAs APDs have been conducted in the past, their waveband overlaps with that of Si, which makes it hard to compete with Si APDs in many applications [15,16]. Despite this issue, AlGaAs APDs played a vital role in the research and development of APD technology. After the commercialization of Si APDs, a second major type of commercialized APD was made of InGaAs with InP or InAlAs multipliers, and their wavebands range from 1–1.7 μm [17,18]. The major advantages of InGaAs APDs are as follows: (1) several applications were found, such as,# free-space optical communication networks, photon counting in quantum computing, range-finding, and lidar imaging; (2) sensitive to the “eye-safe” signals, the wavelengths of which are beyond 1.4 μm. To compete with the InGaAs APDs in the SWIR range, InAs and antimonide compounds were recently demonstrated. Moreover, there are also findings that InAs and antimonide compound-based APDs have applications in the MWIR range [19,20]. For InAs, its band structure is similar to that of HgCdTe, which has noiseless avalanche gain. As for antimonide compounds, their avalanche properties are better than InP or InAlAs multipliers. CdZnTe/HgCdTe APDs were sensitive from the spectral region of SWIR to long-wavelength infrared (LWIR), which had been demonstrated in an airborne differential absorption lidar system [21,22].

Currently, there are several types of distance measurement techniques, including time of flight (TOF), triangulation, time of sight (TOS), phase shift, etc. Among all these techniques, the TOF method is one of the most widely used and straight-forward solutions. To keep the budget low, one should choose the common type of lasers, and these lasers should supply high power in short pulses, thus giving the receiver a strong return signal. For the near-IR lasers that operate in the range from 850 nm to 940 nm, they did not meet the requirements of being “eye-safe”. On the other hand, adverse environmental conditions (such as fog, rain, desert dust, humidity, oceanic aerosol, etc.) make the detection extremely challenging due to the severe scattering and absorption effects between the laser and ambient environment. To deal with this problem, the operation wavelength moves towards 1.55 μm (despite the “eye-safe” issue, a wavelength of 1.31 μm might also be available due to less scattering), which is both “eye-safe” and has less scattering [23,24]. Technically, it is therefore raising the spectral region requirements from the detection terminal (SWIR region).

Current APDs technology operating in the SWIR region is based on InGaAs/InP materials, which have several problems, such as expensive fabrication processes, smaller wafer sizes, high afterpulsing effects, long dead-times, high dark count rates (DCRs), low operation temperatures, etc. Group IV materials have the advantages of tunable band structure, larger wafer size, low cost, and compatibility with Si CMOS technology, which are naturally easier to transfer for mass volume production [25,26]. To extend the spectral wavelength range of group IV APDs, tremendous efforts have been made. A significant breakthrough has been achieved in Ge semiconductor material, whose bandgap difference is only 136 meV. Several strategies were proposed to tune the band structure of Ge, including heavy n-type doping, tensile strain engineering, the incorporation of a group IV Sn (Pb) element into Ge, and their combination technique. It has long been verified that GeSn alloys are able to enhance the spectral absorption coefficient at wavelengths of 1.55 μm and 2 μm, indicating that GeSn is also an effective absorber for group IV APD [27,28,29,30].

**Figure 3:**
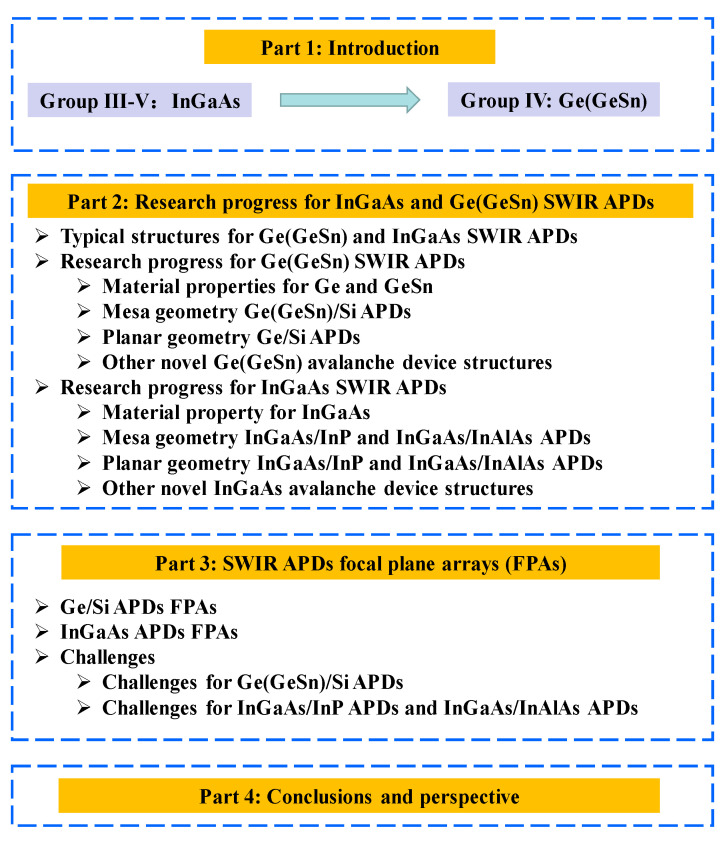
Outline of this review article.

To the best of our knowledge, there are no review articles about APDs made of both Ge (GeSn) and InGaAs materials [31,32,33,34]. The objective of this review paper is to give researchers a full understanding of recent advancements in materials for APDs and the rapid development towards SWIR for future LiDAR systems. The scaffold of this paper is shown in Figure 3. In Section 2, we focus on the recent research progress for Ge (GeSn) and InGaAs SWIR APDs for mesa and planar geometry, and other novel avalanche device structures. In Section 3, we present the advances of SWIR APDs focal plane arrays (FPAs) and the technology challenges and perspectives.

## 2. Research Progress for Ge(GeSn) and InGaAs SWIR APDs

### 2.1. Typical Structures for Ge(GeSn) and InGaAs SWIR APDs

**Figure 4:**
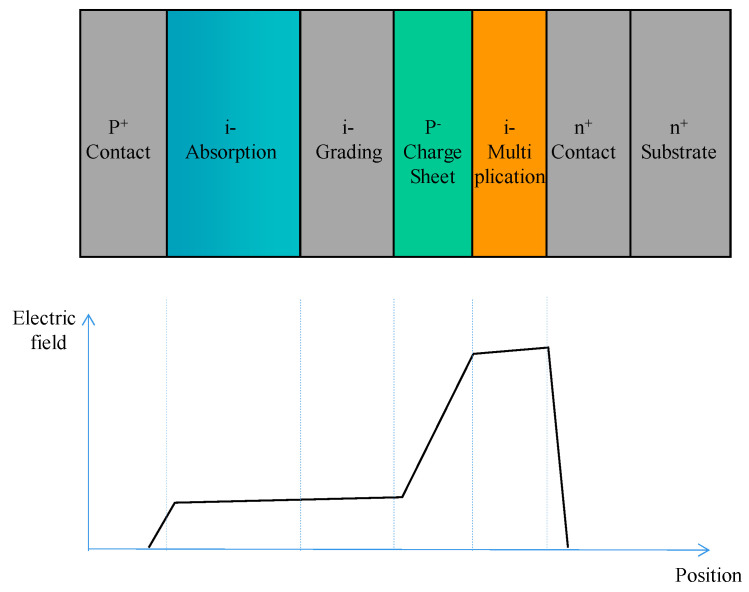
Typical schematic diagram and electric profile in the SAM APD.

**Figure 5:**
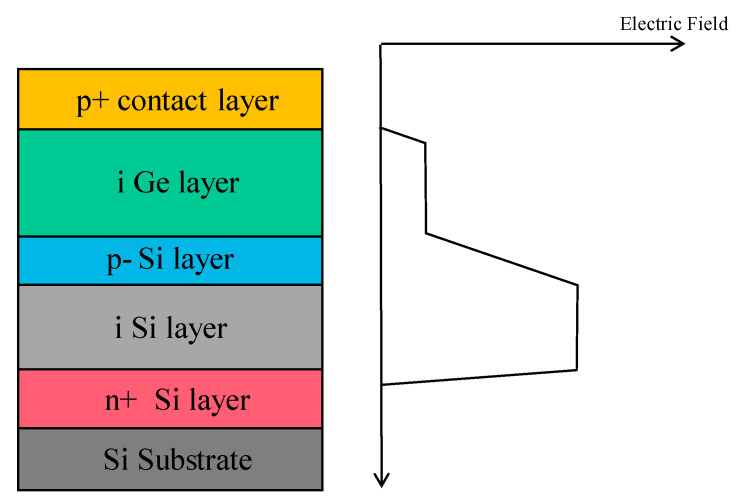
Schematic diagram and electric field distribution of SACM Ge/Si APD.

To achieve high-performance APDs, they should have a large multiplication factor (M), a low excess noise factor (F), and a high gain-bandwidth product (GBP). Thus, separation absorption and multiplication (SAM) structures were proposed, in which only light was absorbed in the absorption region and one carrier type was transported into the multiplication region. Thus, M, F, and GBP were considered. In addition, the electric field should be low enough to avoid band-to-band tunneling in the Ge (or GeSn) and InGaAs materials. Figure 4 shows the typical schematic diagram and electric profile of the SAM APDs [35]. For the case of Ge/Si APDs’ separation absorption, researchers usually use the separation absorption, charge, and multiplication (SACM) structure. The schematic diagram for the SACM Ge/Si APD and its electric field distribution is shown in Figure 5 (GeSn is similar to this structure) [36].

**Figure 6:**
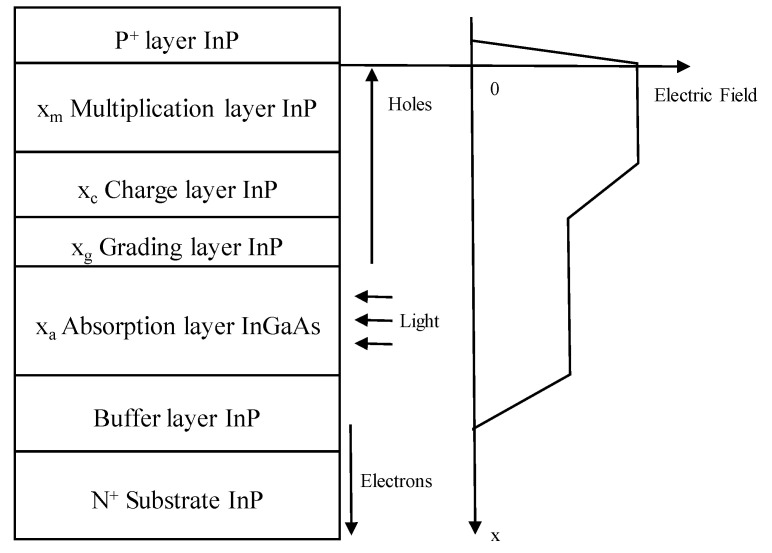
Schematic diagram and electric field distribution of separate absorption, grading, charge, and multiplication (SAGCM) InGaAs/InP APDs.

**Figure 7:**
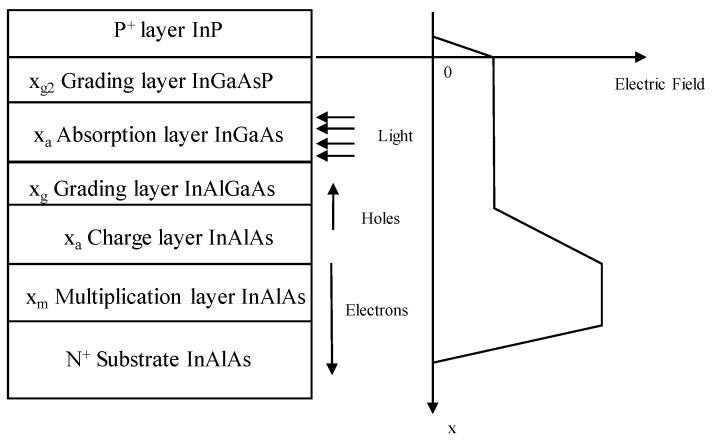
Schematic diagram of separate absorption, grading, charge, and multiplication (SAGCM) InGaAs/InAlAs APDs and its electric field distribution.

For InGaAs/InP APDs, the schematic diagram and its electric field distribution were as follows (Figure 6) [37]. Schematic diagram and electric field distribution of InGaAs/InP APDs were shown in Figure 7 [37].

To the best of our knowledge, both InGaAs/InP APDs and InGaAs/InAlAs APDs are commercially available. However, Ge/Si APDs are also a promising technology to achieve high-performance detection due to their low cost and compatibility with the Si CMOS process.

### 2.2. Research Progress for Ge (GeSn) SWIR APDs

**Figure 8:**
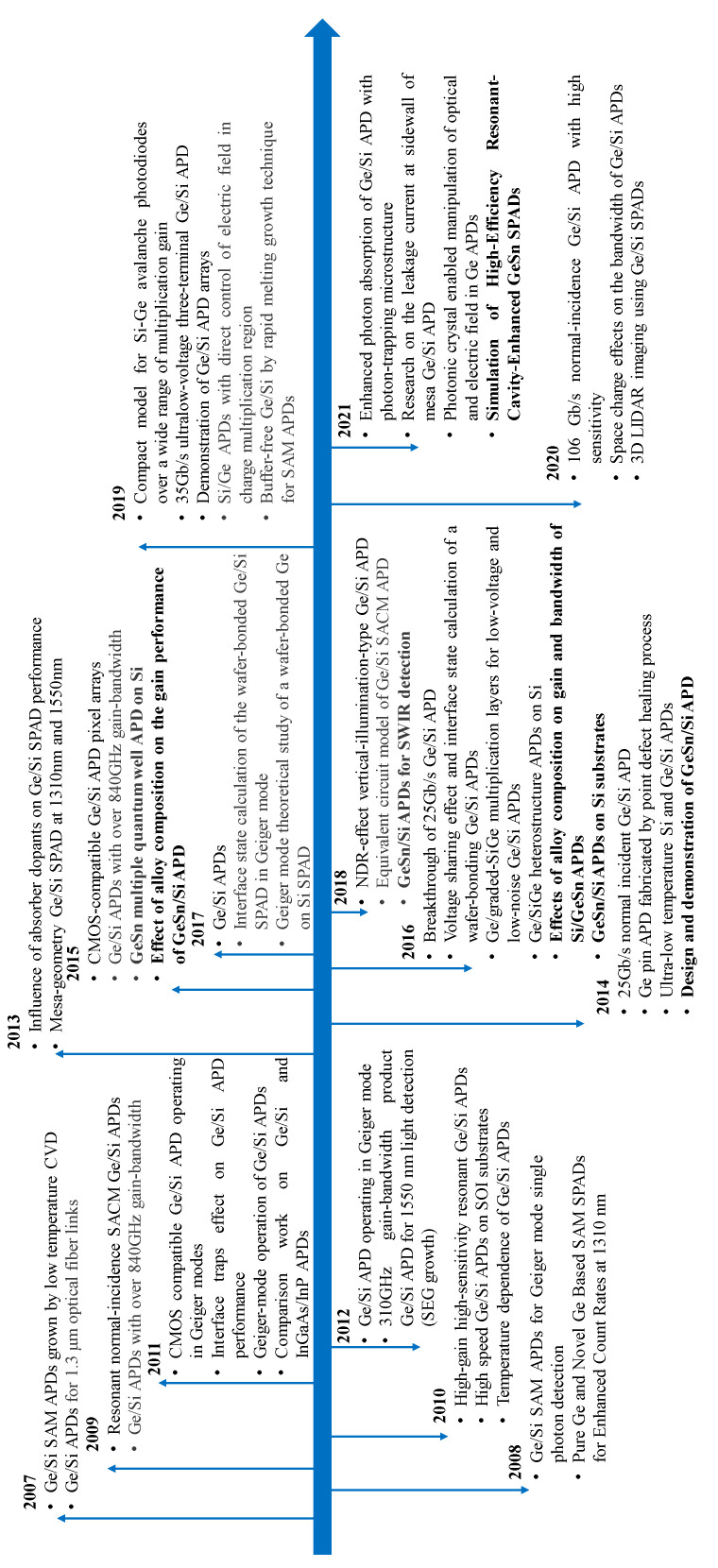
Research development of Ge(GeSn) APDs.

So far, several types of growth techniques, such as MBE [38,39,40,41], magnetron sputtering [42,43], and CVD [44,45], have been used to grow Ge (GeSn) materials. Although Si APD exhibits high performance (high detection efficiency and low dark count probability), its spectral region is limited by the bandgap properties. To extend the operation wavelength, InGaAs or Ge were used as absorbers, and Si was used as a multiplication layer. The reason for using Si as the multiplication region is its low excess noise factor, which is favorable for avalanche breakdown. For the benefits of the Ge absorber, including that Ge was able to grow on large wafer-size Si platforms using CVD technology, it was compatible with traditional CMOS technology, it had good absorption properties with the wavelength cutoff of 1.6 μm at 300 K, it was easily integrated with CMOS circuits, etc. As is shown in Figure 8, both linear mode and Geiger mode Ge/Si APDs were achieved experimentally. To extend the light detection wavelength range, Sn was incorporated into the Ge matrix. Due to the growth technique limitation, only linear mode GeSn APDs were achieved, and GeSn SPADs are still in the simulation stage. Based on the geometry, both mesa-type and planar-type Ge/Si APDs were proposed. However, only mesa-type GeSn APDs were reported, and there is still no experimental demonstration of the planar-type GeSn APDs.

#### 2.2.1. Material Properties for Ge and GeSn

Different from Si, the band structure of Ge differs from Si conduction band arrangement. The energy bandgap difference between the direct bandgap and indirect bandgap of Ge is only 136 meV, and its band structure can be modulated via N^+^-type doping engineering, tensile strain engineering, and Sn-based alloy engineering. As noted earlier, Sn is a group IV element, which is a borderline material. Generally, there are two types of tin allotropes (including gray tin and white tin); gray tin (α-Sn) will transfer to white tin (β-Sn) at near room temperature, and their energy difference is extremely small. α-Sn exists in the form of a diamond structure and is more stable. However, β-Sn structure is distorted from the ideal diamond structure. To form the ideal (Si)GeSn alloys, we expect to incorporate diamond structure α-Sn into (Si)Ge crystals, and no phase transformation occurs (white tin). From the band structure of α-Sn, the lowest conduction band and highest valence band all touch the zone center, indicating the band gap of α-Sn is exactly zero (temperature and pressure are not able to change this special nature) [46,47].

**Figure 9:**
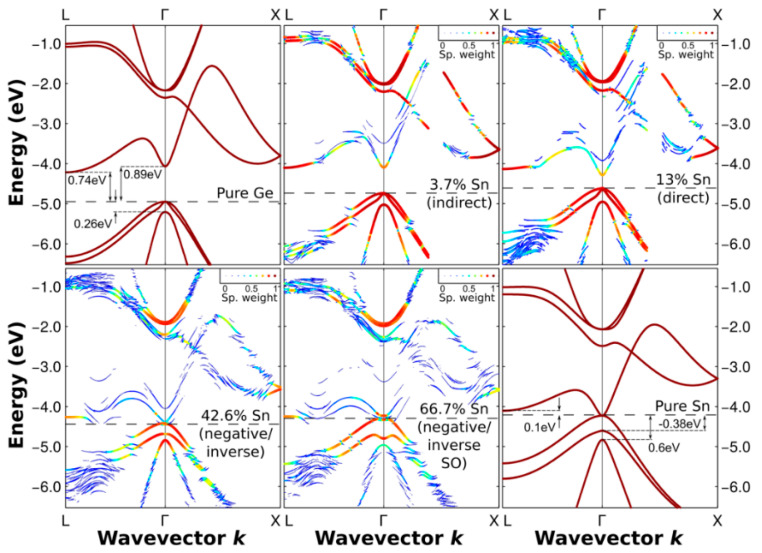
Band structures of bulk Ge and α-Sn (first and last figures, respectively), GeSn materials with various compositions (3.7%, 13%, 42.6%, and 66.7%). The color and point size denote the Bloch spectral weight, and the points with weights lower than 0.05 are neglected. Reproduced with permission from [46], IOP Publishing, 2017.

To extend the light absorption range of pure Ge, Sn-based alloy engineering was considered the most promising routine, which acts similarly to the tensile strain in Ge and also lowers the direct valley below the indirect valley for tunable band structure (Figure 9). More importantly, direct bandgap GeSn can be used as the efficient light absorption layer for Si-based SWIR detectors, which are fully CMOS-compatible with the large-scale OEICs on the low-cost Si platform. Thus, widespread theoretical research on the crossover of direct bandgap GeSn was conducted (Table 1).

**Table 1:** Theoretical study on the material properties of direct bandgap GeSn in terms of institution, theoretical methods, and Sn composition.

Year	Institution	Methods	Sn (%)	Ref.
**1987**	University of Notre Dame	Tight-binding calculations using virtual-crystal approximation (VCA).	>20	[48]
**1989**	Swiss Federal Institute of Technology	Self-consistent ab initio pseudo-potential calculations in VCA.	27–74	[49]
**1999**	Michigan Technical University	First-principles calculation in nonlocal density approximation to density functional theory (GGA) with Becke exchange functional with Ferdew-Wang’s correlation functional.	50	[50]
**2007**	University of Leeds	Charge self-consistent pseudo-potential Xα method.	17	[51]
**2008**	Fudan University	First-principles fully relativistic band structure method and a more accurate approach that considers core-level volume deformation (VD) potential.	25, 50, 75	[52]
**2011**	Stanford University	First-principles calculations using density function theory (DFT) with GAA+U.	3, 6	[53]
**2012**	National University of Singapore	Empirical pseudo-potential method (EPM), 8-band k.p method	0–20.	[54]
**2012**	Shenyang University of Technology	First principles calculations based on norm-conserving pseudo-potentials, density function theory (DFT), and density functional perturbation theory (DFPT).	——	[55]
**2013**	Stanford University	Virtual crystal approximation (VCA) within the framework of the nonlocal empirical pseudo-potential method (NL-EPM).	0–20, 7	[56]
**2014**	ETH Zurich	Empirical pseudopotential method (EPM) along with virtual crystal approximation (VCA).	0–20	[57]
**2015**	Chinese Academy of Science, Institute of Semiconductor	First-principle calculations based on DFT are performed using the Vienna ab initio simulation package (VASP) code. To correct the underestimation of the bandgap, the GGA+U approach with Perdew–Burke–Ernzerhof (PBE) is employed to describe the electron exchange and correlation.	0–20, 8.5	[58]
**2017**	Wroclaw University of Science and Technology	First-principle calculations based on DFT along with Tran and Blaha MBJLDA functions. All calculations have been performed with the all-electron full potential WIEN2k code, which has recently been proven to be one of the most accurate DFT codes.	3.7–66.7	[46]
**2018**	University College Cork	First-principle calculations based on DFT, including Heyd Scuseri Ernzerhof (HSE) hybrid functions, using the Vienna Ab initio Simulation Package (VASP).	6.25	[59]
**2019**	National Chiao Tung University	Nonlocal empirical pseudopotential method (EPM) with modified virtual crystal approximation (VCA), 8-band k.p model.	0–15, 7.1	[60]
**2019**	Nanyang Technological University	Empirical tight binding and ab initio methods, full-zone 30-band k.p model.	0–30, 7.25	[61]
**2020**	George Washington Unviersity	Combining statistical sampling based on the Monte Carlo method and density functional theory (DFT) calculation using the Vienna Ab initio Simulation Package (VASP) based on the projector augmented wave method.	25, 8	[62]
**2020**	Tiangong University	First-principle calculation via the sX-LDA method, using the Cambridge Sequential Total Energy Package (CASTEP) code based on the DFT, The norm-conserving pseudopotentials (NCPP) are used to describe the interactions between the electrons and ionic cores.	0–30, 6.25	[63]
**2021**	University College Cork	First-principle calculations based on Kohn–Sham DFT, Structural relaxations are carried out in the local density approximation (LDA), and electronic band structures are calculated using the Tran–Blaha modified Becke–Johnson (TB-mBJ) meta-generalized gradient approximation (meta-GGA) exchange-correlation potential in order to overcome the band gap underestimation typical of the LDA in the Kohn–Sham formalism.	0–100, 5, 11, 25	[64]

**Figure 10:**
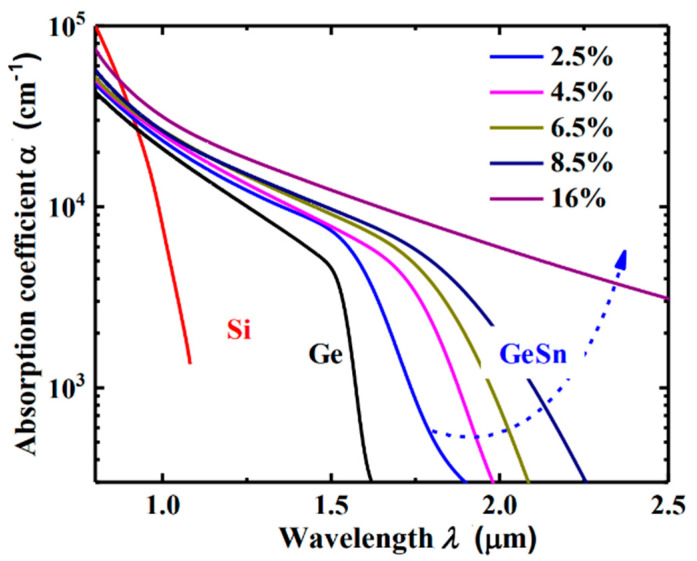
Absorption coefficient of compressive GeSn on Ge with various Sn compositions ranging from 2.5 % to 16 %. Reproduced from [65], open access by NUS Library, 2019.

Many theoretical research works have been conducted to evaluate the material properties of GeSn, especially for the band structure of direct bandgap GeSn. Among all these theory methods, density functional theory (DFT)-based first-principles calculations have been shown to yield an overall good agreement with experimentally observed GeSn band gaps. However, there is still a lack of an in-depth study on the point defect behavior on the GeSn material properties, whether Sn vacancies or Ge vacancies. The existence of a point defect will affect the material properties, thus increasing the leakage current and reducing the quantum efficiency of the GeSn photodetectors. To determine the absorption coefficient of GeSn with various compositions, extensive spectroscopic ellipsometry measurements had been performed for all the samples (Figure 10). GeSn layers were grown on Ge (100) substrate, which suffers from compressive strain.

In the past decade, tremendous efforts had been made on the GeSn photo detection. With a very small incorporation (2%) of Sn into Ge, the absorption coefficient for GeSn is several times higher than that of bulk Ge (Figure 10). In the meantime, the cutoff wavelength for the Ge_0.98_Sn_0.02_ photodetector is sufficient to fully cover all the telecom windows (from O-band to U-band: 1260 nm to 1675 nm) [27]. By incorporating more Sn atoms into Ge, wavelength coverage can be further extended to longer wavelengths. For SWIR detection, incorporation of 10–11% of Sn is necessary to cover the entire SWIR wave band, which is extremely suitable for the thick GeSn layer due to the strain relaxation growth mechanism [28]. Experimental research had demonstrated that the cutoff wavelength was extended to 3.7 μm with more than 20% Sn incorporation [29].

#### 2.2.2. Mesa Geometry Ge(GeSn)/Si APDs

At the initial research stage for Ge(GeSn) APDs, mesa structures are generally acknowledged due to their reproducibility and fabrication simplicity.

**Figure 11:**
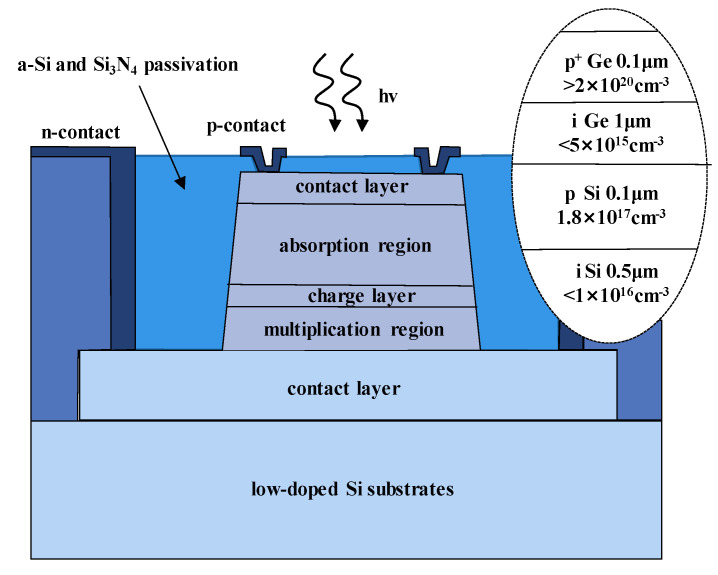
Schematic cross-section of Ge/Si SACM APD.

Y. Kang et al. [66] demonstrated the first mesa-type Ge/Si APDs for 1.31 μm light detection, which are based on the conventional separate absorption charge and multiplication (SACM) structure (Figure 11). Ge and Si are designed as the absorber and multiplier, respectively. To obtain this structure, a commercial CVD chamber was utilized to grow the Si multiplication layer and Ge absorption layer. After the growth, circular mesas were wet-etched. To reduce the threading dislocation densities (TDDs) originating from the Si/Ge interface, an annealing process was carried out in the temperature range of 800 °C–900 °C. Si_3_N_4_ film was deposited as passivation and planarization, which is also used as an anti-reflection coating. The characterization results exhibit a responsivity of 0.54 A/W with the 1.31 μm incident light, dark current densities as low as 237 mA/cm^2^ at 90% breakdown voltage, a breakdown voltage thermal coefficient of 0.05%/°C, and a 3dB-bandwidth of 10 GHz.

**Figure 12:**
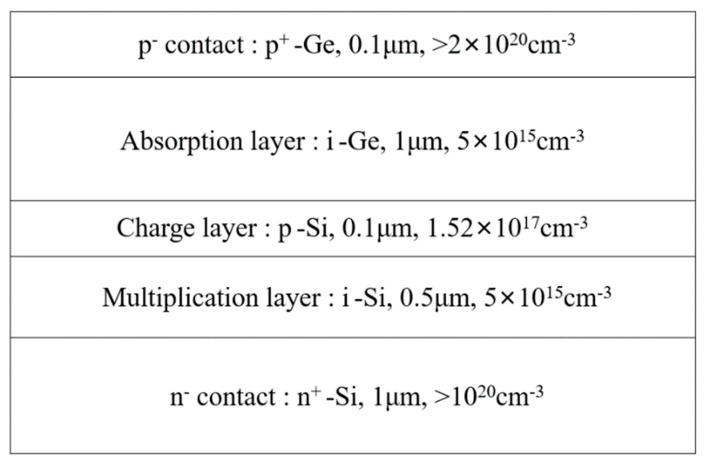
Layer structure for separate-absorption-charge-multiplication (SACM) Ge/Si APD.

Zhiwen Lu et al. [67] demonstrated the first relatively complete single photon counting results for Geiger-mode-operated Ge/Si APD. The thicknesses of the Ge absorption layer and Si multiplication layer are 1 μm and 0.5 μm, respectively (Figure 12). The charger layer is p-type doped (1.52 × 10^17^ cm^−3^) with a thickness of 0.1 μm. Owing to the large lattice mismatch between the p-Si charger layer and the i-Ge absorption layer (4%), considerable traps were generated in the interface, leading to the higher dark current. To characterize the single photon detection efficiency (SPDE) and dark count rate (DCR), a 1.31 μm pulsed laser was employed as the light source (the optical pulse width and pulse repetition rate are 50 ps and 100 kHz, respectively). Experimental results show that an SPDE of 14% and a DCR of 10^8^ Hz were achieved. However, DCR is very high, and the device does not have enough time to recover its bias lever before other DCs are triggered, which will cause the underestimation of DCR at a certain bias.

**Figure 13:**
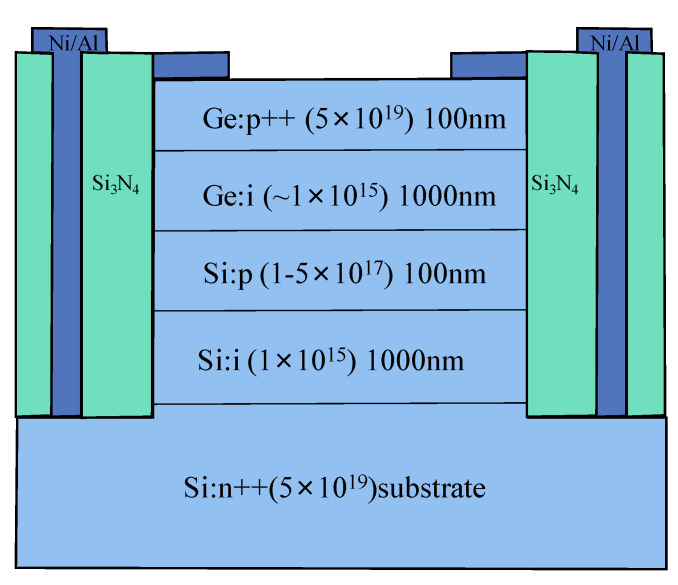
Cross-section schematic of Ge/Si mesa type APD structures (Si_3_N_4_ was utilized for passivation and planarization).

To improve the device’s performance, several modifications had been made to the above-mentioned APD structures (Figure 13). First, the i-Si multiplication region was increased to 1.0 μm, which will raise the voltage difference between the avalanche breakdown voltage and punch-through voltage. Then, the doping concentration for the p-Si charger layer was designed in the range of 1 × 10^17^cm^−3^ to 5 × 10^17^cm^−3^. As punch-through voltage depends merely on the doping concentration of the charger layer, one can alter the voltage difference by changing the doping level. Especially at low temperatures, small voltage differences will remarkably lower the SPDE for APD at 1.31 μm and 1.55 μm. To characterize the low-temperature performance, APD devices were mounted in the liquid nitrogen (LN_2_)-cooled cryostat. Both pulsed semiconductor lasers were employed to evaluate the SPDE at wavelengths of 1.31 μm and 1.55 μm [68]. With the illumination of a 1.31 μm pulsed laser, low temperature (100 K) SPDE is approximately 4% at 10% excess bias. However, low temperature SPDE (at the wavelength of 1.55 μm) was measured to be 0.15% at 6% excess bias, which is mainly due to the reduced absorption edge for Ge at low temperatures. At the temperature of 150 K, DCRs of 10^6^–10^7^ Hz were clearly observed, which is still several orders of magnitude higher than InGaAs/InP planar SPADs (10^2^–10^3^ Hz). These high DCRs were attributed to the high dark currents due to the high defect level in the Ge absorption layer and narrow bandgap of Ge. Moreover, sidewall surface recombination was also responsible for the high dark current.

**Figure 14:**
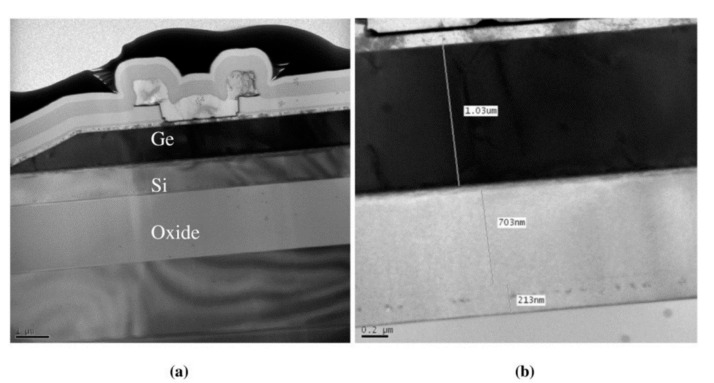
TEM images of (**a**) mesa-type Ge/Si APD by SEG growth; (**b**) total layer thickness. Reproduced from [69], OSA Publishing, open access, 2012.

Different from previously reported mesa-type Ge/Si APD, Ning Duan et al. [69] adopted selective epitaxial growth (SEG) to form the Ge absorption layer and the Si multiplication layer. On the one hand, SEG Ge growth was able to reduce the device processing complexity; on the other hand, it can also prevent the mesa edge breakdown. Growth procedures for SACM Ge/Si APD are as follows: (I) high-resistivity (100) SOI substrates with 220 nm Si layer and 2000 nm buried oxide; (II) bottom n-type Si contact formation: As ion implantation; (III) SEG growth of 700 nm Si multiplication layer at 600 °C; (IV) p-type charge layer formation: implanting B into the as-grown Si; (V) SEG growth of 1000 nm Ge absorption layer: including 50 nm SiGe buffer, 50 nm Ge seed layer growth at 350 °C, and high temperature Ge growth at 550 °C (Figure 14). With proper design, the responsivity and gain-bandwidth of the SEG-grown mesa-type Ge/Si APD at 1550 nm are 12 A/W and 310 GHz, respectively. One important point for selective growth integration is pattern dependency. The growth rate (and composition) can be altered due to chip layout. This means that the uniformity issue may arise over the process wafer and has to be tackled [70,71,72,73].

**Figure 15:**
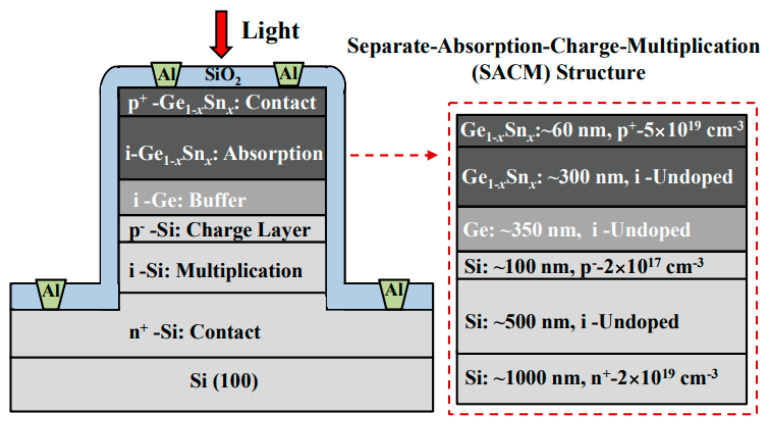
Schematic for first demonstration of SACM GeSn APD with Sn composition up to 5%. Reproduced with permission from [74], IEEE, 2014.

**Figure 16:**
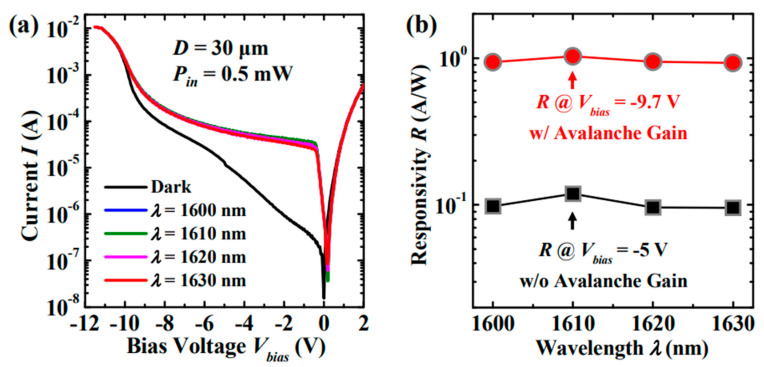
(**a**) I-V_bias_ characteristics of GeSn/Si APD under the illumination of 1600 nm, 1610 nm, 1620 nm, and 1630 nm incident laser light; (**b**) relationship between responsivity and wavelength at −5 V and −9.7 V. Reproduced with permission from [74], IEEE, 2014.

Although Ge was used as the absorber to realize Ge/Si APDs, the absorption coefficient of Ge becomes very low at wavelengths beyond 1.6 μm, which makes Ge/Si APDs unsuitable for imaging in the wavelength range from 1.6–3 μm. With the incorporation of Sn into Ge, an enhanced absorption coefficient was observed, and its absorption range was also extended beyond 1.6 μm. Due to its enhanced absorption range and absorption coefficient, GeSn was designed as the absorber for SACM GeSn/Si APD (Figure 15). To characterize the GeSn/Si APD with a mesa size of 30 μm, incident laser wavelength and laser power were employed as 1600–1630 nm and 0.5 mW, respectively. Figure 16 shows the I-V_bias_ characteristics and responsiveness for the GeSn/Si APD. It is clearly observed that the I-V_bias_ curves stay almost the same with various incident laser wavelengths (1600–1630 nm). However, responsivity increases significantly with the bias voltage of −9.7 V owing to the internal gain (Figure 16b).

**Figure 17:**
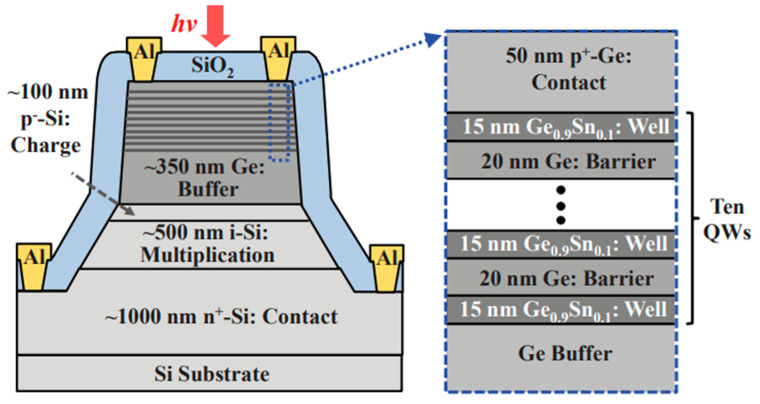
Cross-sectional schematic of GeSn/Ge MQWs APD with SACM structure. Reproduced with permission from [75], IEEE, 2015.

Different from the SACM APD with bulk GeSn absorber, the GeSn/Ge MQWs absorber has the following advantages: (1) increase the critical thickness; (2) MQWs’ structure is favorable for light absorption, emission, and modulation, which is also helpful for future optoelectronic integrated circuits (OEICs). Thus, photons are absorbed in the GeSn/Ge MQWs structure and electrons are generated; charge multiplication occurs in the intrinsic Si layer (Figure 17). With the 10% Sn incorporation into GeSn/Ge MQWs, the wavelength cutoff was further extended above 2 μm.

**Figure 18:**
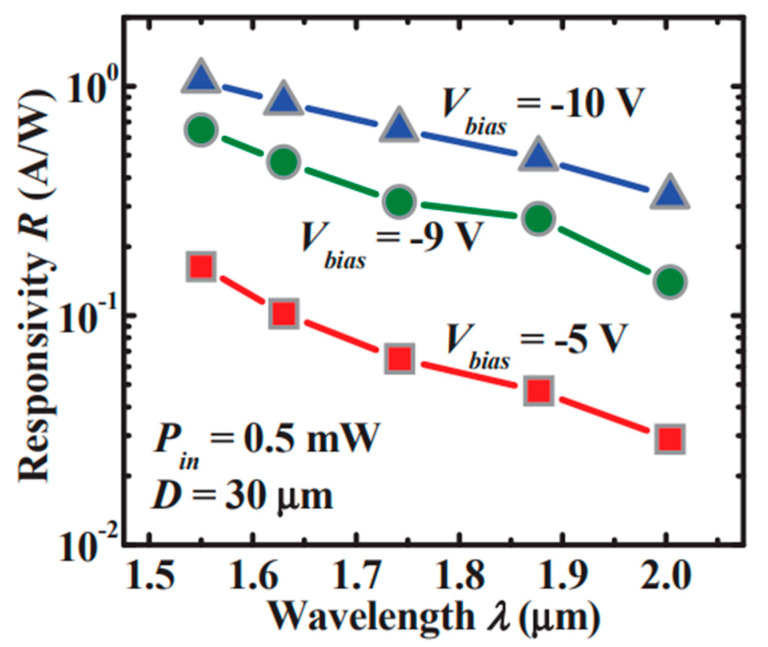
Optical responsivity for GeSn/Ge MQWs APD is biased at different voltages. Reproduced with permission from [75], IEEE, 2015.

Figure 18 illustrates the wavelength-dependence responsivity of GeSn/Ge MQWs APD. To carry out the optical response measurement, two types of light sources (including tunable lasers and distributed feedback laser diodes) were used. At each incident wavelength (1510 nm, 1630 nm, 1742 nm, 1877 nm, and 2003 nm), GeSn/Ge MQWs APDs were biased at −5 V, −9 V, and −10 V. At the wavelength of 2003 nm, responsivities for GeSn/Ge MQWs’ APD biased at the voltages of −5 V and −10 V are 0.029 and 0.33 A/W, indicating avalanche multiplication occurs and peak avalanche gain is evaluated to be ~15 when biased at -10 V. In addition, the thermal coefficient of the breakdown voltage is calculated to be 0.05% °C^−1^, which is smaller than InGaAs/InP APDs, suggesting GeSn/Ge MQWs APD is less dependent on the temperature.

**Figure 19:**
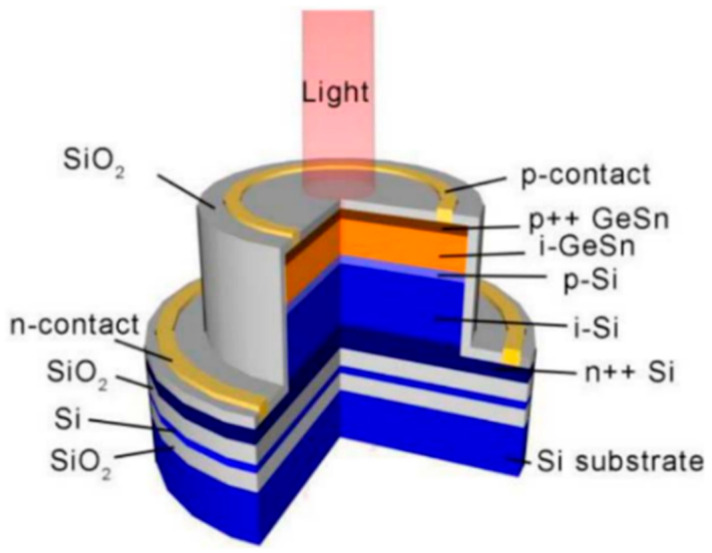
Proposed RCE GeSn SPAD structure with GeSn absorber and Si multiplication layer. Reproduced with permission from [76], IEEE, 2021.

Despite the rapid development of GeSn photodetectors, GeSn SPADs have not been demonstrated yet. To achieve high SPDE, low DCR, and high operation temperatures in GeSn SPADs, Qimiao Chen et al. [76] proposed resonant-cavity-enhanced (RCE) GeSn SPADs operating at 1.55 μm and 2 μm, respectively (Figure 19). Major design considerations were as follows: (I) To decrease the DCR, the SACM structure was employed so as to avoid the band-to-band tunneling in the GeSn absorption layer; (II) photon absorption probability can be greatly improved by sandwiching the GeSn absorber and the Si multiplication layer with vertical cavity, thus increasing the SPDE (Sn compositions for the 1.55 μm and 2 μm light absorbers were 3% and 10%, respectively); (III) the electric field intensity in the Si multiplication layer is the highest in the whole layer structure, and the electric field distribution should also be uniform; (IV) the electric field in the GeSn absorber needs to be moderate, thus photon-generated electrons are able to drift into the high-electric field Si multiplication layer and make the contribution to the SPDE; (V) high background doping concentrations and high defects in the GeSn absorber cause a negative effect on SPADs, which is important for high-performance SPADs. With proper design, SPDE for GeSn SPADs was predicted to be 80% at the wavelength of 1.55 μm, which features RCE GeSn SPADs are a promising candidate for high efficiency single photon detection in the SWIR region.

#### 2.2.3. Planar Geometry Ge/Si APDs

**Figure 20:**
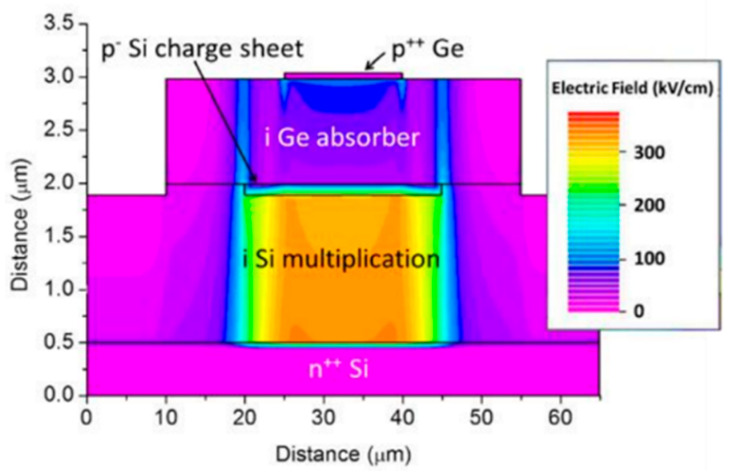
Electric field distribution for Ge/Si SPAD at 5% excess bias above the breakdown (the thickness of the Ge absorber is 1 μm). Reproduced from [77], ROS Theses Repository, open access, 2019.

As researchers mentioned, planar geometry avoids the sidewall contribution to the dark current and can further decrease the DCRs; thus, Ge APD has the potential to operate at higher temperatures, and SPDE might also be significantly improved. To examine the electric field distribution in the whole device structure, a TCAD simulation study for the planar geometry Ge/Si APDs was performed. Figure 20 shows the optimal electric field distribution due to the following considerations: (I) the electric field in the i-Ge absorber is low at breakdown, so photo-generated electrons can drift to the i-Si multiplication layer more efficiently; (II) the electric field in the i-Si multiplication layer is high, which is helpful for the impact ionization of electrons; (III) the electric field was confined into the center of the Ge SPAD, preventing the sidewall effects.

**Figure 21:**
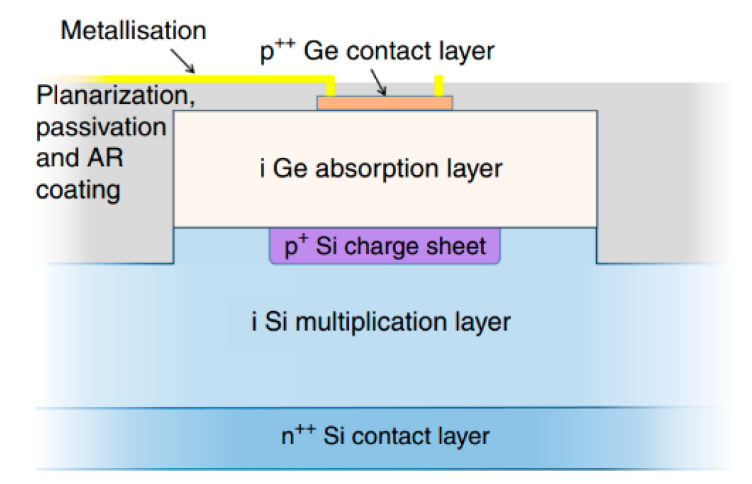
Cross-section diagram of a planar Ge/Si SPAD structure with an i-Ge absorption layer, a p^+^-Si charge layer, and a i-Si multiplication layer. Reproduced from [77,78], ROS Theses Repository, and Springer Nature, open access, 2019.

**Figure 22:**
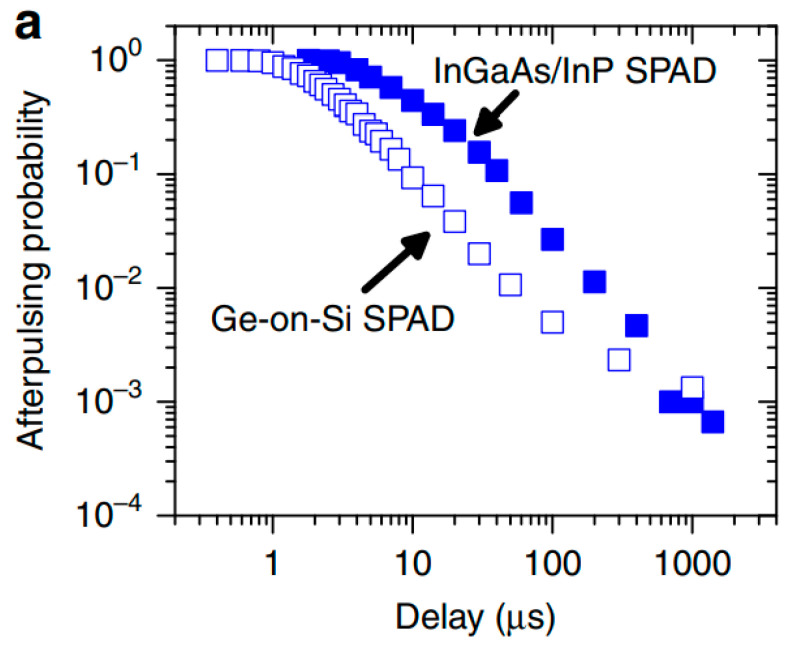
Comparison of afterpulsing probabilities vs. gate delay time between planar Ge/Si SPAD (100 μm diameter) with commercial InGaAs/InP SPAD (25 μm diameter). Reproduced from [78], Springer Nature, open access, 2019.

In 2019, Peter Vines et al. [77] demonstrated a new generation of planar Ge/Si SPAD for SWIR operation (Figure 21). Compared with the mesa-type, planar geometry was able to avoid the sidewall recombination effect, which is helpful for DCR reduction. Before the 1.3 μm laser radiation, SPADs were cooled down to a low temperature for SPDE, DCR, and jitter measurements. At 100 K, SPDE was improved to 26%, which is several times higher than previous reported mesa-type SPADs (4%). Moreover, DCR was three orders of magnitude lower than mesa-type results. After increasing the temperature to 125 K, SPDE for planar geometry Ge SPADs (38%) is comparable to the commercial InGaAs/InP SPADs. Based on Beer–Lambert’s law, low temperature (125 K) SPDE can be further improved to be 55% with a 2 μm Ge absorption layer. The jitter full width at half maximum (FWHM) is 310 ps, which can be optimized by decreasing the device diameter. To check the afterpulsing mechanism for planar geometry Ge/Si SPAD, afterpulsing probability was evaluated at a temperature of 125 K, which features 20% of the InGaAs/InP SPAD (Figure 22). A similar trend was also observed at 150 K. Moreover, the noise equivalent power (NEP) of 1.9 × 10^–16^ W/Hz^1/2^ at T = 78 K is a 50-fold improvement on previously reported Ge-on-Si SPADs. From the planar Ge/Si SPAD results, smaller diameter devices with low TDD and thick Ge absorption layers will allow the device to work at higher temperatures (high SPDE at the wavelength of 1.55 μm), lower DCRs, and lower afterpulsing probabilities.

To further decrease the DCRs of 100 μm diameter planar geometry for Ge/Si SPAD, device diameters were fabricated to be 26 μm and 50 μm. Device performance for the Ge/Si SPAD with different diameters was summarized as follows: in terms of temperature, SPDE, DCRs, Jitter, and NEP (Table 2). It was found that an extremely low DCR was achieved for the 26 μm diameter detector, indicating excellent sensitivity for planar geometry Ge/Si SPAD. Although the maximum SPDEs of the 26 μm and 50 μm devices were 10% lower than those of the 100 μm device, jitter and the NEP for the 26 μm device were 157 ± 10 ps and 9.8 × 10^−17^ WHz^−1/2^, respectively. The 26 μm diameter device shows record-low NEP, which suggests planar geometry has the potential to achieve low-cost and high-speed Ge/Si SPAD arrays in the SWIR spectral region. Compared with the commercial InGaAs/InP SPAD, the Ge/Si SPAD features lower DCRs and shorter data acquisition times.

To further improve the SPDE for planar geometry Ge/Si SPAD, it is important to evaluate the effect of Ge absorber thickness. Generally, thicker absorbers contribute to better absorption and more photogenerated carriers. Based on Lambert–Beer’s law, less than 50% of 1.31 μm light is absorbed in the 1 μm Ge absorber. To improve the absorption efficiency, it is essential to increase the Ge absorber thickness. As is estimated by the researchers, 75% and 88% of 1.31 μm light are absorbed within 2 μm and 3 μm Ge absorbers, respectively.

**Figure 23:**
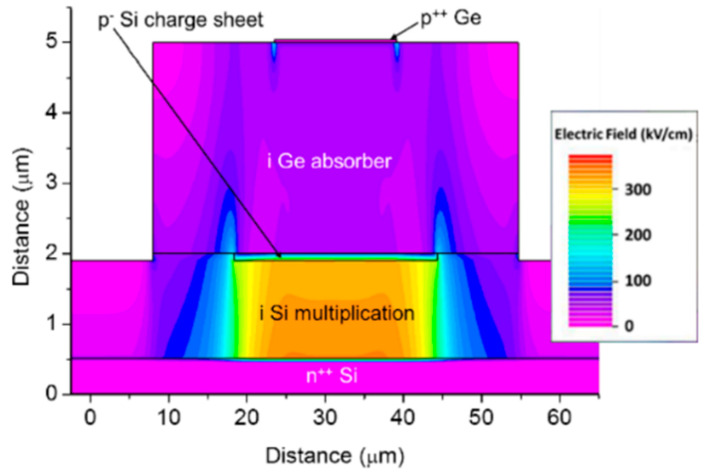
Electric field distribution for Ge/Si SPAD at 5% excess bias above the breakdown (the thickness of the Ge absorber is 2 μm). Reproduced from [77], ROS Theses Repository, open access, 2019.

**Figure 24:**
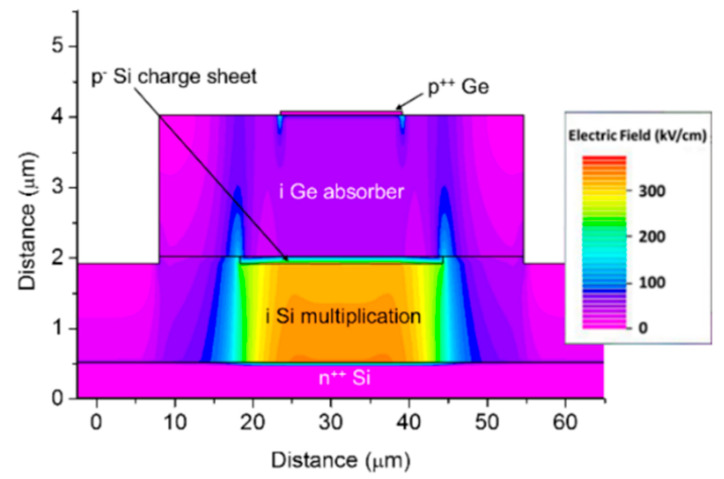
Electric field distribution for Ge/Si SPAD at 5% excess bias above the breakdown (the thickness of the Ge absorber is 3 μm). Reproduced from [78], Springer Nature, open access, 2019.

From Figure 23 and Figure 24, electric field distributions for Ge/Si SPAD with 2 μm and 3 μm thick absorbers were clearly observed. Compared with Ge/Si SPAD with a 1 μm absorber, the electric field near the sidewall is higher for the 3 μm absorber, indicating that the dimensions of Ge/Si SPAD still require further design optimization.

#### 2.2.4. Other Novel Ge(GeSn) Avalanche Device Structures

The emerging two-dimensional (2D) layered materials have proven to be a promising candidate for photodetection due to their excellent properties, including ultrathin structure, thickness-dependent band structure, dangling bond immunity, and high light absorption. Another type of candidate material for photodetectors could be 2D crystals, especially graphene. The disadvantage of such detectors is their low response time [80,81].

**Figure 25:**
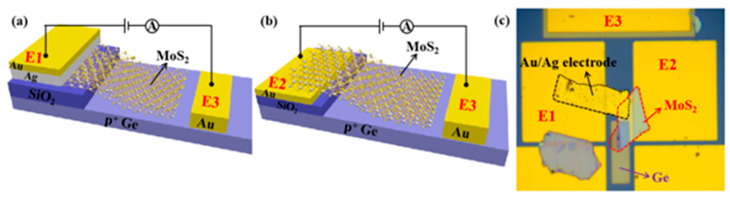
Schematic diagram of MoS2/Ge heterojunction with (**a**) Ag electrode, (**b**) Au electrode, and (**c**) optical image of MoS2/Ge heterojunction. Reproduced from [82], Optica Publishing, open access, 2022.

Efforts have been made to develop 2D/Ge van der Waals heterostructures for broadband photodetection (Figure 25). Recently, Yuan et al. [82] demonstrated a novel avalanche photodetector based on p-Ge/n-MoS2 van der Waals heterojunction, which shows that the heterojunction with an Ag electrode exhibits a better rectification effect and lower breakdown voltage than the device with an Au electrode. The device shows a maximum responsivity of 170 and 4 A/W under 532 and 1550 nm illumination, respectively. The device with high optical detection performance under both visible and infrared illumination provides a new, novel, and promising path for broadband photodetection.

**Figure 26:**
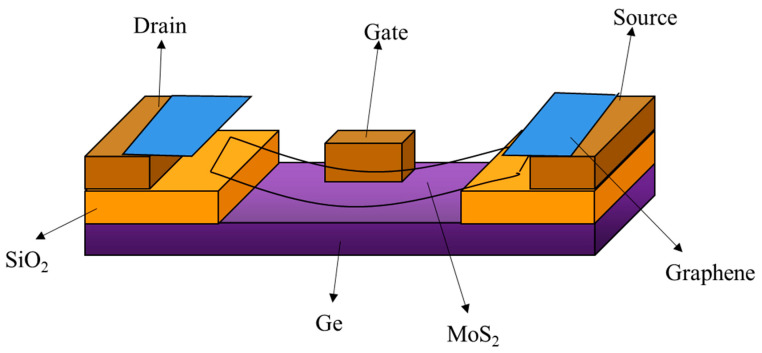
Schematic diagram structure of MoS_2_/Ge heterostructure JFETs.

The 2D/3D heterojunctions can also be appropriately applied to junction field-effect transistors (JFETs), which operate by changing the depletion region of the p-n junction. Gate-tunable and photoresponsivity behaviors of JFETs based on 2D/3D heterojunctions are demonstrated in ref [76]. The JFET shows a low sub-threshold swing of ≈88 mV/dec and a high on/off current ratio of ≈10^5^. By modulating the gate voltage, the device can reach a peak positive responsivity of 66 A/W under visible illumination at 532 nm. In contrast, the device exhibits a modifiable negative responsivity behavior under IR illumination at 1550 nm due to the combined effect of different polarity currents in MoS_2_ and Ge (Figure 26) [83]. These results provide a new strategy for the development of novel 2D/3D optoelectronic heterostructures that have good potential as multifunctional optoelectronic units.

**Figure 27:**
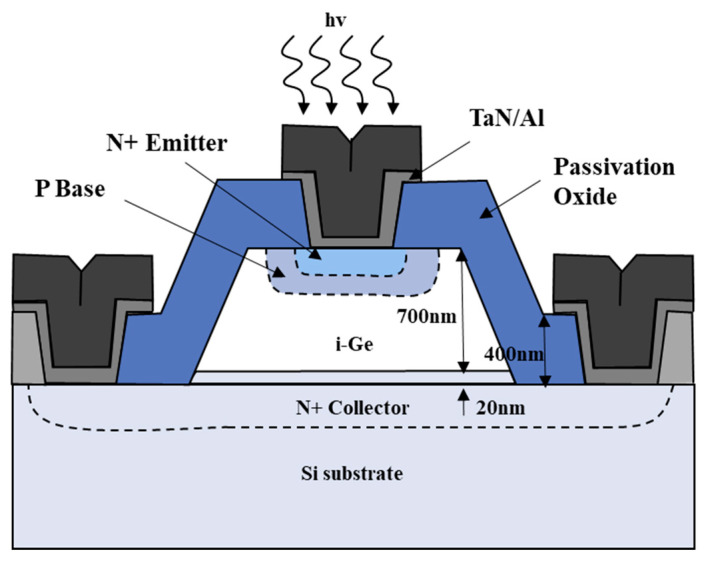
SEM image and schematical diagram of a Ge phototransistor.

For many years, APDs were considered an attractive option for digital fiber-optic communications. However, induced impact ionization requires high operating voltages (typically greater than 8 V) and can introduce considerable noise, which hinders its practicality. Another option is to use phototransistors (PTs) to provide high responsivity and bandwidth while biasing at low voltages. Some group III–V compound alloy materials, such as (GaAl)As/GaAs and InP/InGaAs, have been widely used to fabricate such phototransistors. Recently, the development of group IV-based semiconductor materials has also started to receive increasing attention, and active efforts are being made to realize high-speed PTs [84]. Ang et al. [85] fabricated floating-base germanium phototransistors with the N/P/i-Ge/N configurations by using a low-temperature selective Ge epitaxy process (Figure 27). The current gain induced by transistor action allows the device to achieve large photocurrent and optical response enhancement at a low operating bias compared to conventional p-i-n Ge photodetectors. An impressive 2.0 A/W responsivity is obtained at 1.55 μm optical illumination at −1.0 V.

**Figure 28:**
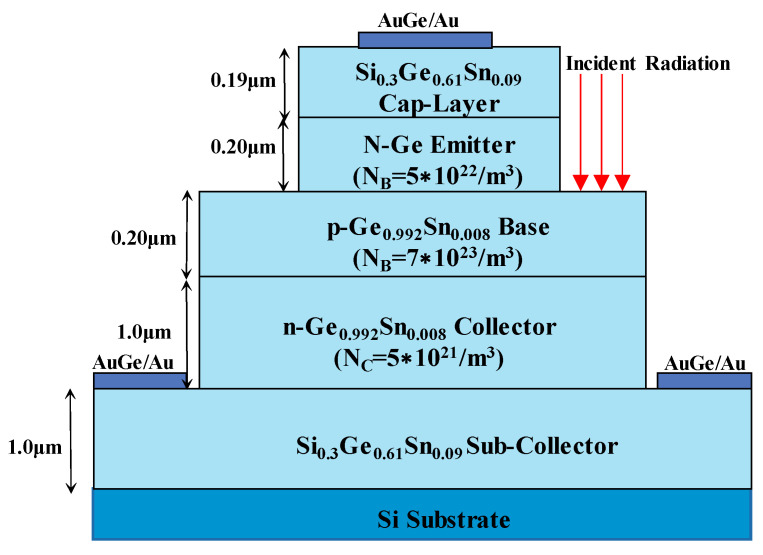
Schematic diagram for a front-side illuminated Ge–GeSn–GeSn hetero phototransistor.

Photonic devices using group IV semiconductors on Si platforms have been of great interest for decades due to the indirect nature of the band gap of Si materials. However, there is a large lattice mismatch between the two materials, which makes the growth of Ge/GeSn on Si substrates a problem. In the last 15 years, the situation has improved dramatically with the successful growth of GeSn and the discovery of practical chemical vapor deposition (CVD) routes. The formation of high-quality GeSn films and GeSiSn alloys directly on Si substrates can be achieved. The addition of a few percent of substitutional Sn to Ge can effectively narrow the direct band gap of Ge and extend the wavelength range for absorption to more than 1550 nm. Ordinary p-i-n PDs have no internal optical gain, and APDs with internal optical gain have the disadvantages of high operating voltage and high noise. GeSn phototransistors (PTs) are considered a useful alternative to normal PDs because they have internal optical gain while operating at low voltage and without excess noise. In 2015, the performance of a front-side illuminated Ge–GeSn–GeSn hetero phototransistor grown on a Si platform was demonstrated [85]. The terminal currents, current gain, optical gain, and responsivity of the HPT at 1.55 µm were calculated. People find that the values are comparable with those in InGaAs/InP systems, and the values for GeSn-based HPTs are even higher for lower values of base doping and base width. The proposed structure can be grown on a silicon platform, which allows it to be used as a viable alternative to InGaAs-based detectors in the C and L-band for fiber optic communications (Figure 28).

### 2.3. Research Progress for InGaAs SWIR APDs

**Figure 29:**
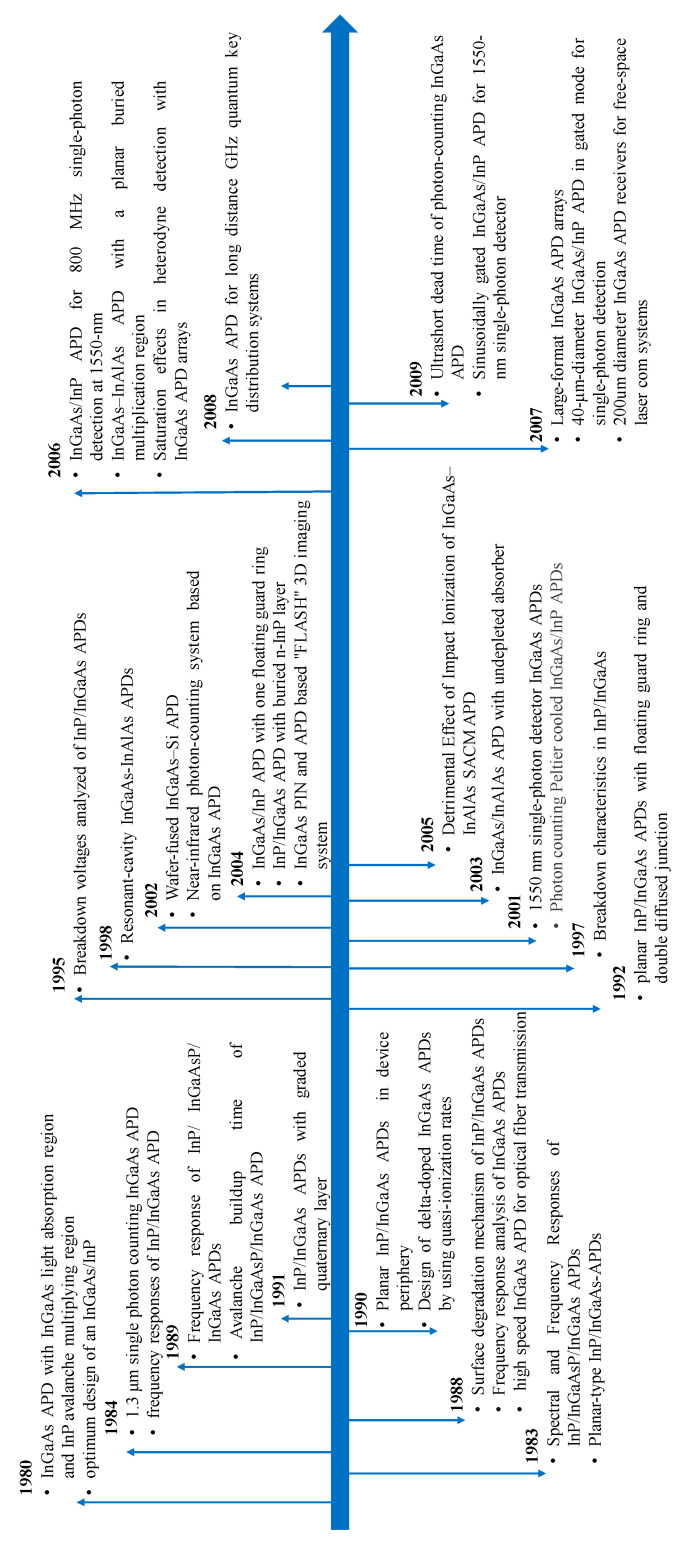

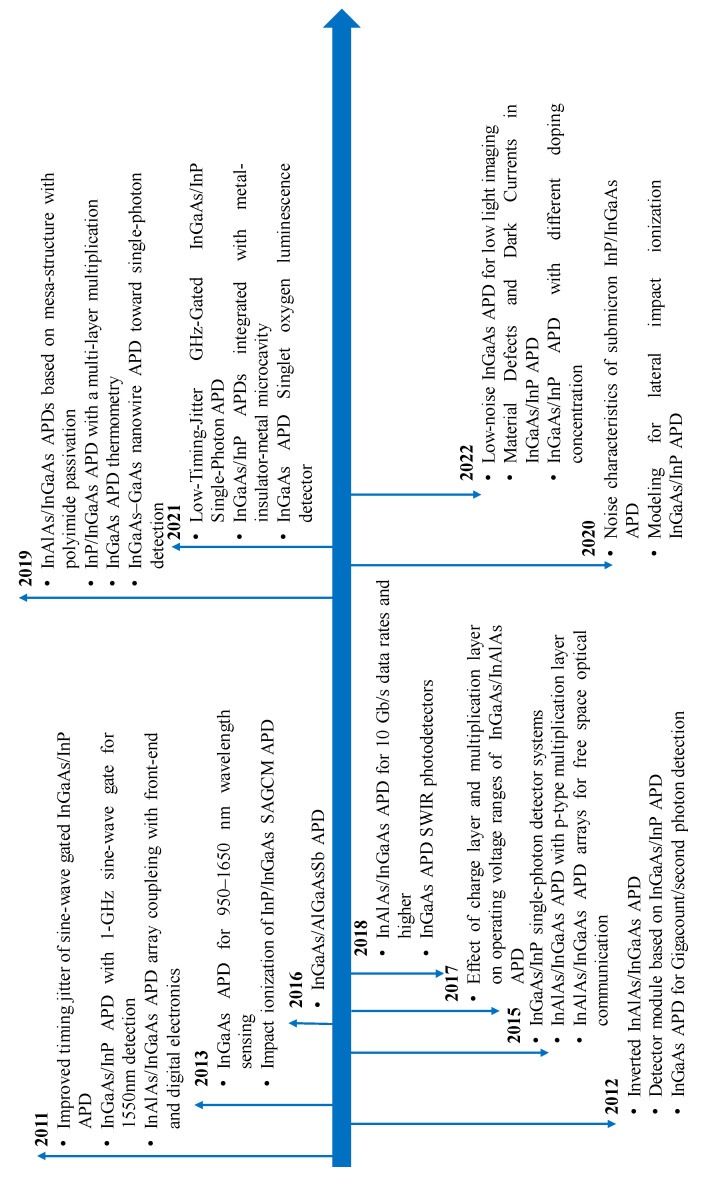
Research development of InGaAs APDs.

Up until now, there have mainly been two techniques to grow the InGaAs/InP APDs, including molecular beam epitaxy (MBE) and metal organic chemical vapor deposition (MOCVD). Different from MBE, MOCVD can achieve a high growth rate at a lower vacuum, which is more suitable for industrial large-scale growth. Compared with the Ge/Si APDs, In_0.53_Ga_0.47_As/InP APDs possess superior performances in the SWIR range (0.9–1.7 μm), which covers the 1550nm eye-safe LiDAR (Figure 29). As one of the most promising eye-safe APDs, SAGCM structure InGaAs/InP APDs were generally acknowledged. Due to the small lattice mismatch between In_0.53_Ga_0.47_As and InP (0.0424%), In_0.53_Ga_0.47_As and InP were employed as the absorption region and multiplication region, respectively. To avoid carrier accumulation at the heterostructure interface and alleviate the energy discontinuity between the In_0.53_Ga_0.47_As and InP interfaces, the InGaAsP grading layer was introduced. Due to the lattice match between InGaAs and InAlAs, InAlAs were also utilized as the multiplication region for the InGaAs/InAlAs APDs, which feature better higher gain at the low dark current level. Therefore, InAlAs is gradually replacing the InP multiplication region owing to its superior ionization coefficients, which leads to reduced excess noise. At the same time, InAlAs has a wider bandgap, which is also helpful for the reduction of tunneling current.

#### 2.3.1. Material Property for InGaAs

**Figure 30:**
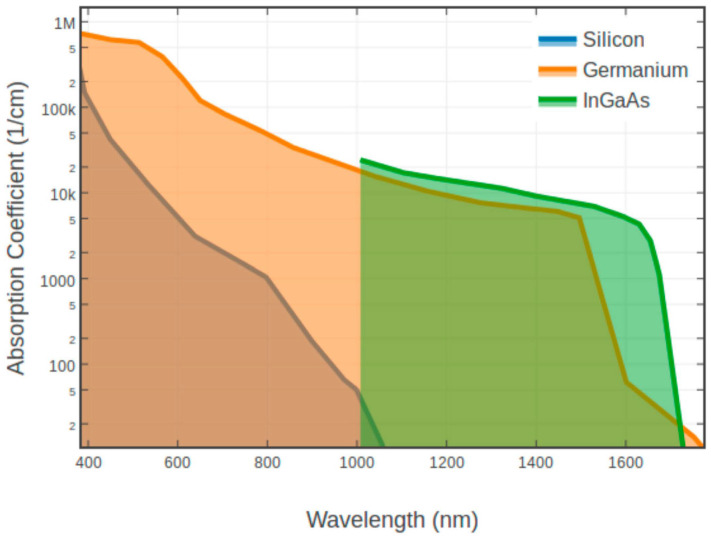
Comparison absorption characteristics of Si, Ge, and InGaAs. Reproduced from [86], open access by White Rose eTheses Online, 2020.

The optical and electronic properties of ternary InGaAs can be aligned by varying the ratio of InAs and GaAs. The cutoff wavelength of ternary In_0.53_Ga_0.37_As is approximately 1.7 μm, which is well lattice matched with the InP substrate, which has been widely used for SWIR detection. To extend the wavelength cutoff, more indium should be introduced, but this leads to a larger mismatch with InP, thereby generating defects and degrading the crystal quality. Accordingly, In_0.53_Ga_0.37_As is the superior choice to form the high-quality SWIR absorber, especially for the 1550 nm eye-safe LiDAR application. Compared with the Ge absorber, In_0.53_Ga_0.37_As has superior absorption characteristics at 1550 nm over Ge (Figure 30). To this point, InGaAs APDs are more suitable for 1550 nm eye-safe light detection than Ge/Si APDs [86]. It is highly expected that GeSn/Si APDs should be a cost-effective routine to compete with the InGaAs APDs for this application. However, research for the GeSn/Si APDs is still in the preliminary stages, which makes the InGaAs APDs more competitive in the market at the present.

#### 2.3.2. Mesa Geometry InGaAs/InP and InGaAs/InAlAs APDs

**Figure 31:**
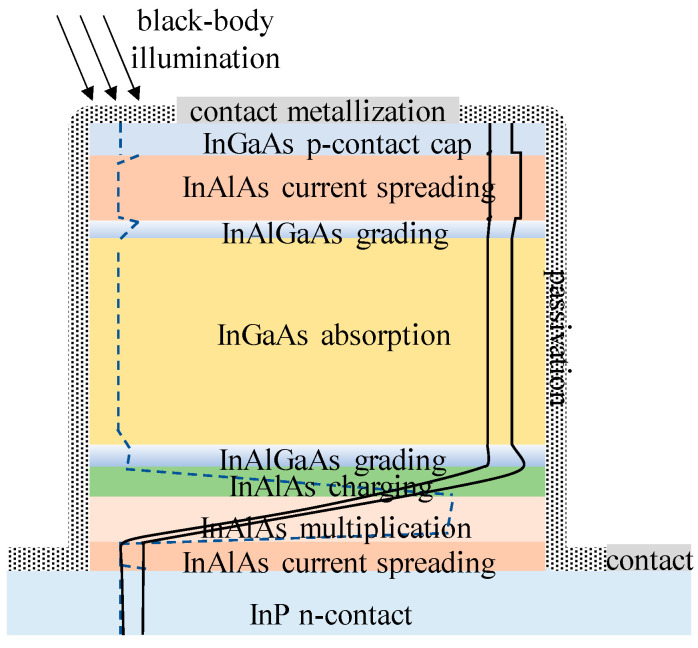
Schematic diagram of InGaAs/Inyalas SAGCM APDs with calculated band-edge profile.

**Figure 32:**
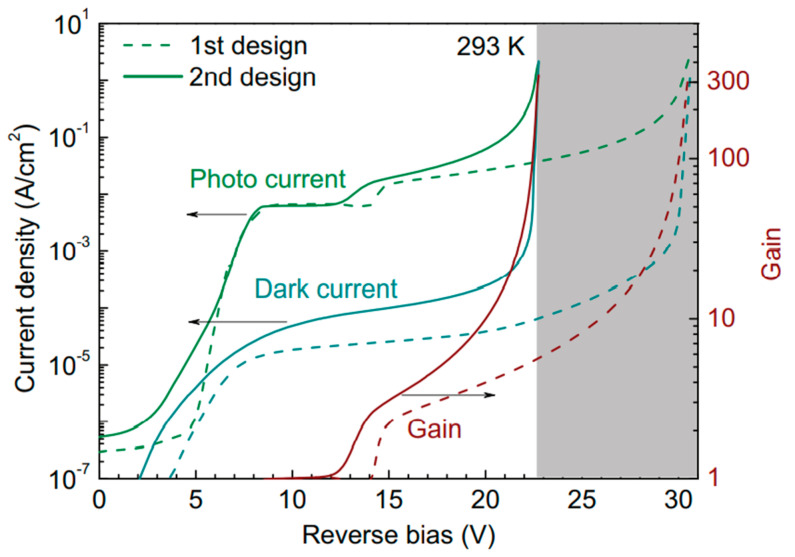
Dark- and photo-current characteristics (left axis) and multiplication gain (right axis) of different charge-layer designs. Reproduced with permission from [87], WILEY-VCH, 2016.

P.Kleinow et al. [87] have grown the InGaAs/InAlAs SAGCM structure on an n+-InP substrate with a combination of a p-type grading layer and charge layer by the MBE system. It had been confirmed that the doping level in the charge layer affects the punch-through voltage (V_pt_), break voltage (V_b_), and multiplication (M) gain performance. Therefore, special consideration should be given to compromise both the V_pt_ and the V_b_ when designing the charge doping level. Despite the charge doping level, p-type doping for the grading layer will reduce the conduction-band barrier between the absorber and multiplication region. Based on the above-mentioned considerations, two designs were proposed. In the first design, it contains a 50 nm undoped InAlGaAs grading layer followed by a 150 nm InAlAs charge layer; in another design, there is also p-type doping in the InAlGaAs grading layer. The main difference between these two designs is the conduction-band barrier height, which is considerably reduced in the second design. Thus, the punch-through voltage was lowered from 10 V (first design) to 8.5 V (second design) (Figure 31). The improved design features a lower dark current density of 1.7 × 10^−4^A/cm^2^ at 20 V bias voltage (Figure 32).

**Figure 33:**
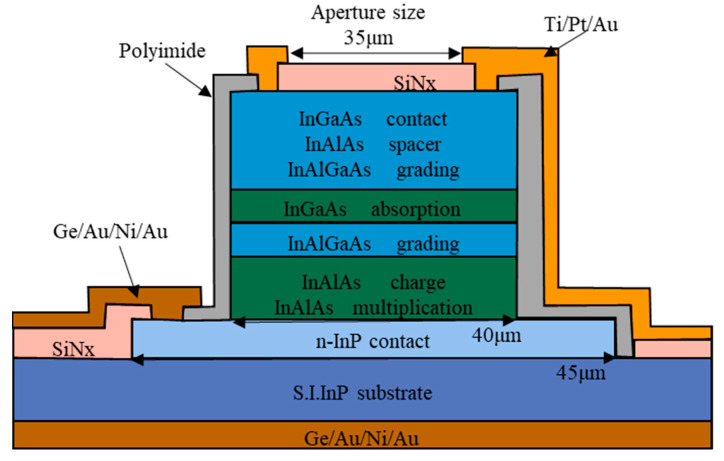
Cross-sectional diagram and top view image for the mesa geometry front-side illuminated InGaAs/InAlAs SAGCM APDs.

Jae-Sik Sim et al. [88] designed and fabricated the mesa geometry front-side illuminated InGaAs/InAlAs SAGCM APDs (Figure 33). Under the 1550 nm laser illumination, V_ph_ and V_br_ were 10 V and 33 V, respectively. Responsivity was evaluated to be 0.8 A/W@1550 nm, and low dark current (2 × 10^−6^cm^−2^) was observed at the multiplication gain of 10.

**Figure 34:**
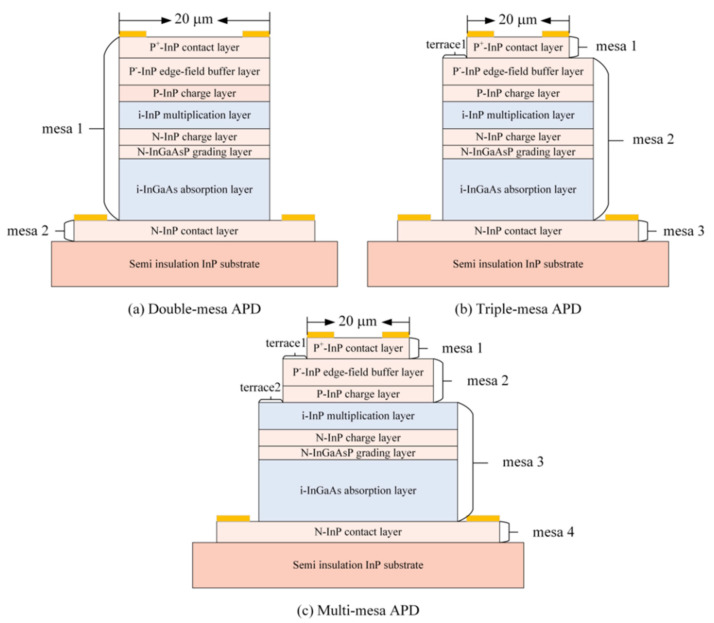
Cross-sectional diagram for the mesa geometry of InGaAs/InAlAs SAGCM APDs with different mesa. Reproduced from [89], AIP Publishing, open access, 2022.

Junqin Zhang et al. [89] investigated the surface leakage current mechanism for the InGaAs/InAlAs SAGCM APDs with various mesas. A typical cross-sectional diagram for InGaAs/InAlAs SAGCM APDs with double-mesas, triple mesas, and multi-mesas were demonstrated in Figure 34. For the double-mesa structure, mesa 1 is for the P-type InP contact layer, P-type InP charge layer, I-InP multiplication layer, N-type charge layer, N-InGaAsP grading layer, and I-InGaAs absorption layer. However, mesa 2 is only for the N-InP contact layer (Figure 34a). For the device structure with triple mesas, mesa 1 and mesa 3 are for the P+-InP contact layer and the N-InP contact layer, respectively. Mesa 2 is for the other parts of the APDs, which include the P-type InP charge layer, the I-InP multiplication layer, the N-type charge layer, the N-InGaAsP grading layer, and the I-InGaAs absorption layer (Figure 34b). In the multi-mesa APD, there are four mesas, mesa 1 and mesa 4 are for the P+-InP contact layer and the N-InP contact layer, respectively. Mesa 2 is for the P-InP charge layer and mesa 3 is for the I-InP multiplication layer, N-InP charge layer, N-InGaAsP grading layer, and I-InGaAs absorption layer (Figure 34c). Finally, they concluded that the main reasons for the generation of sidewall leakage current in the mesa type APDs are as follows: (I) surface charges in the InP multiplication layer, (II) surface recombination centers in the InGaAs absorption layer, (III) intrinsic semiconductor defects. It is found that sidewall leakage current was affected by the mesa geometry, suggesting that multi-mesa APD has been lowest sidewall leakage current level.

**Figure 35:**
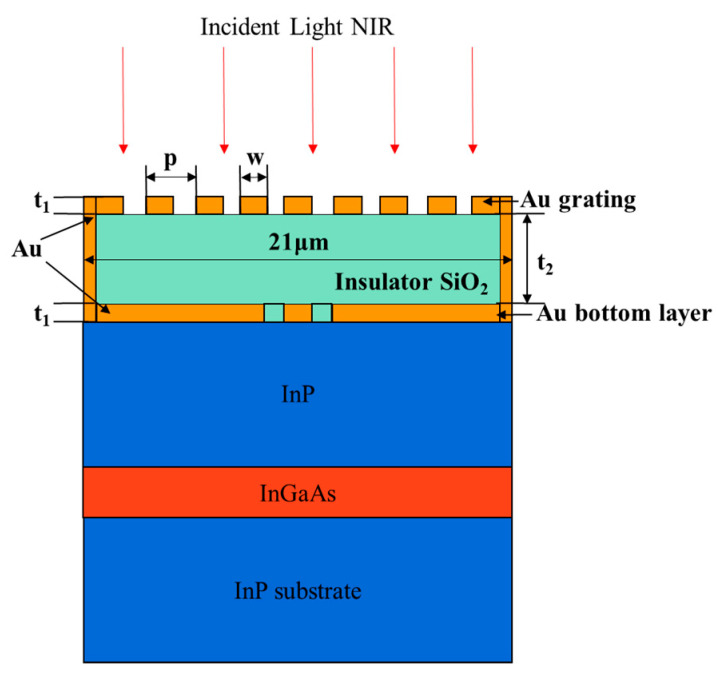
Schematic diagram of InGaAs/InP APDs with metal-insulator-metal (MIM) optical microcavity and cross-sectional of MIM structure.

For the traditional InGaAs/InP SAGCM APD structure, the dark current is higher due to the inherent material defects, which contribute to the lower signal-to-noise ratio (SNR). To solve this problem, reducing mesa the size is an effective method, but it comes at the expense of the optical response. Figure 35 shows the schematic diagram of the InGaAs/InP APD with MIM microcavity, MIM microcavity is directly fabricated on the APD, which includes a top Au grating layer, a middle SiO_2_ insulator, and a bottom Au double slit layer [90]. The main innovation for this structure is that the MIM microcavity can collimate and focus the incident light and have a higher optical transmittance, which makes it able to achieve the lower dark count rates by reducing the APD size but without sacrificing the optical response and quantum efficiency. Compared with the traditional APD, the SNR of the MIM-APD is twice that of the traditional one, and the 3 dB bandwidth is increased by 49%.

#### 2.3.3. Planar Geometry InGaAs/InP and InGaAs/InAlAs APDs

Nowadays, the planar geometry is the mainstream for InGaAs/InP-based SAGCM APDs in commercial products owing to its superior surface leakage current suppression efficiency compared to the mesa geometry. Generally, a high-field p-n junction is required to form the planar geometry of InGaAs APDs, which is achieved by thermal diffusion and/or ion implantation. Since this process is critical for the multiplication region, charge region, and absorption region. After the diffusion process, the p+- region is not an ideal rectangle structure but a circle geometry, which will cause unwanted problems. Hence, a floating guard ring (FGR) structure is used to overcome these problems. To reach a higher surface electric field, p-n junctions require FGR structures (such as single FGRs, double FGRs, deep FGRs, etc.), which will improve the parasitic capacitance and increase the process complexity. Especially for the smaller size APDs, size and location of the FGRs play an important role in the device’s operation.

**Figure 36:**
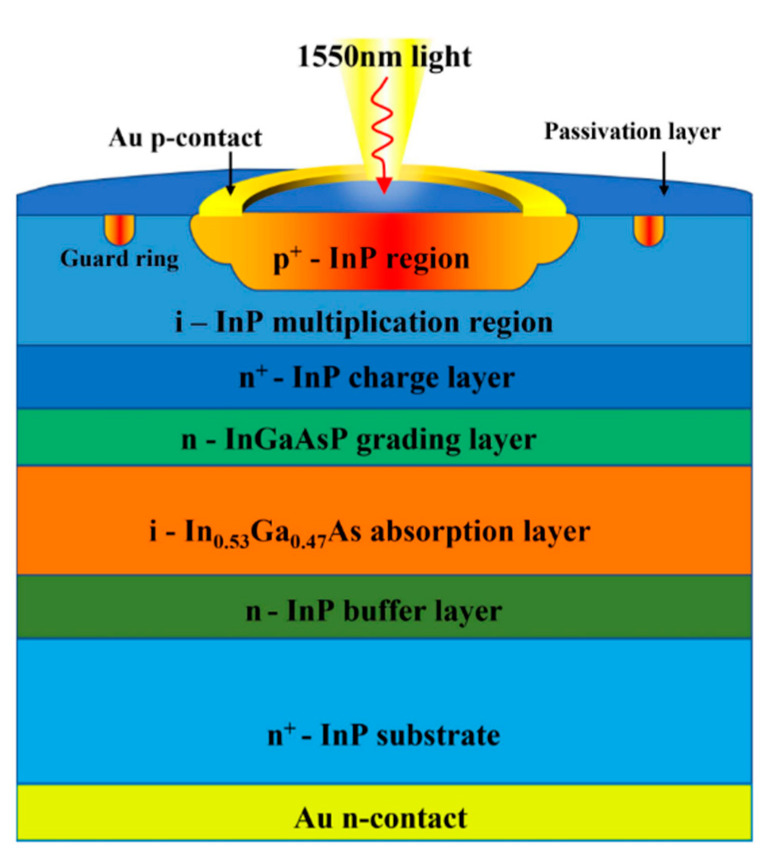
Schematic illustration for InGaAs/InAlAs SAGCM APDs. Reproduced with permission from [91], IEEE, 2022.

**Figure 37:**
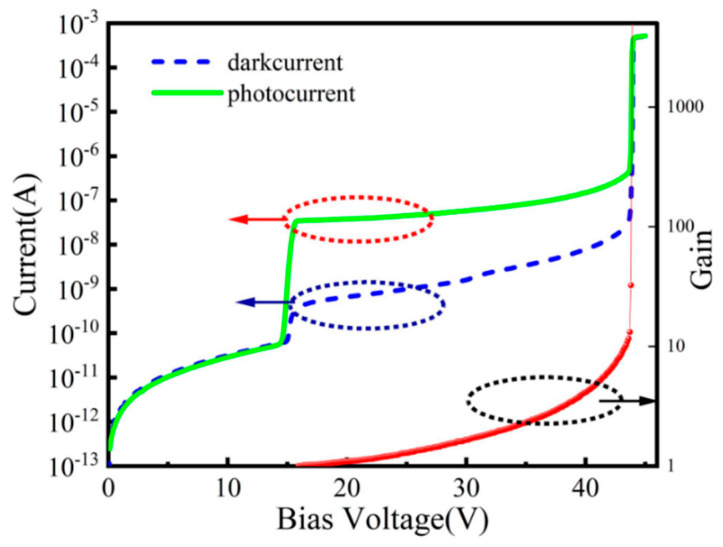
I-V and gain characteristics of the InGaAs/InP SAGCM structure. Reproduced with permission from [91], IEEE, 2022.

Zilu Guo et al. [91] grew the InGaAs/InP SAGCM structure on an InP substrate by the MBE system (Figure 36), and deep low-temperature PL spectra were employed to clarify the effect of the material point defect on the dark current of the APDs. From the PL result, there is deep energy level defect in the InGaAs absorption region, which is most likely produced by the point defect from the MBE growth procedure. Then, planar InGaAs/InP APD is fabricated by the Zn diffusion method and guard-ring process, and the intrinsic InP region below the Zn diffusion region is regarded as the multiplication region. Under 20 nW of 1550 nm light illumination, I–V and gain characteristics show that punch-through voltage (V_pt_) and breakdown voltage (V_b_) are 16 V and 44 V, respectively (Figure 37). In the case where APDs operate in a range from 16 V to 95% of the breakdown voltage, the photo current starts to increase from 3.47 × 10^−8^ to 1.93 × 10^−7^ A, and the dark current also increases with the reverse voltage (3.89 × 10^−10^ to 1.09 × 10^−8^ A). It was also found that the multiplication gain for the dark current is higher than that of the photo current with the increased electric field, which is mainly due to the material defects. It was also confirmed by the theoretical simulation results.

**Figure 38:**
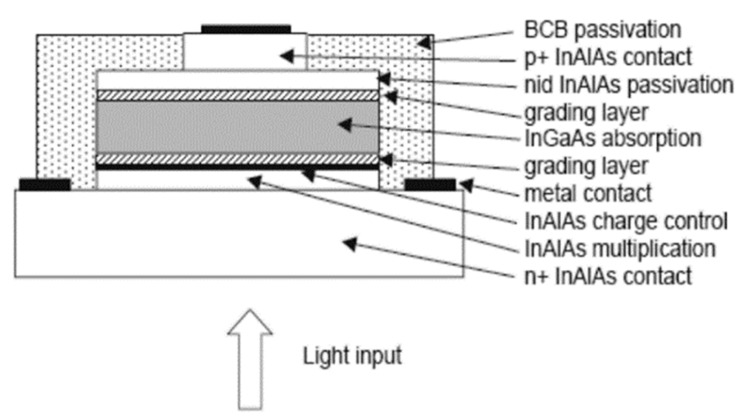
Schematic illustration for the backside illuminated InGaAs/InAlAs SAGCM APDs. Reproduced with permission from [92], IEEE, 2006.

B. F. Levine et al. [92] reported a novel backside-illuminated InGaAs/InAlAs APD with a planar buried multiplication region, which is shown in Figure 38. Compared with other standard APDs, this structure has several advantages: (I) both InAlAs multiplication region and the InAlAs charge region were in-situ doped in the growth chamber, (II) the fabrication process was greatly simplified, (III) there was good wafer uniformity and high reproducibility, etc. Experimental result shows that GBP for this structure is as high as 150 GHz.

**Figure 39:**
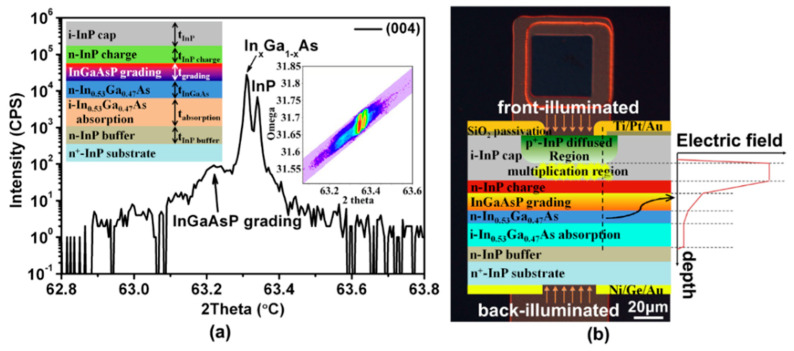
(**a**) HR-XRD scan and device layer structure; (**b**) double side illuminated SAGCM InGaAs/InP APDs with electric field distribution. Reproduced with permission from [93], Elsevier, 2022.

**Figure 40:**
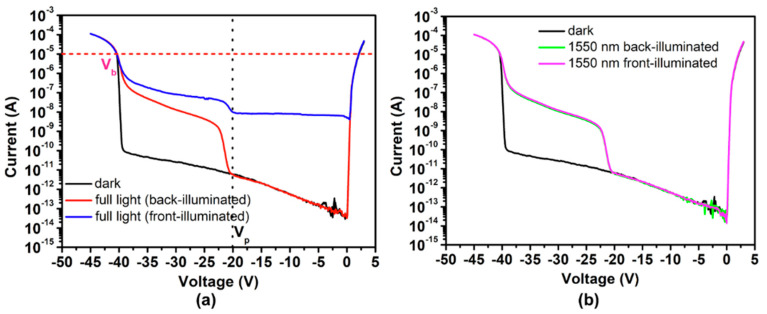
Electrical properties of the double side illuminated SAGCM InGaAs/InP APDs under (**a**) a full-spectrum light source and (**b**) 1550 nm illumination. Reproduced with permission from [93], Elsevier, 2022.

Yiren Chen et al. [93] used the MOCVD reactor to grow In_0.53_Ga_0.47_As/InP structures on a n^+^-InP substrate. From the HR-XRD results, two main diffraction peaks of In_0.53_Ga_0.47_As and InP were clearly observed; another gradient peak corresponds to the InGaAsP grading layer (Figure 39a). To compare the performance contrast between the planar In_0.53_Ga_0.47_As/InP APDs with front- and back-illumination (Figure 39b), both a full-spectrum light source (a tungsten halogen lamp) and a 1550 nm wavelength light source were utilized. The electrical properties (dark current vs. photocurrent as a function of bias voltage) are shown in Figure 40. When the InGaAs/InP APDs were back-illuminated by the full light, photocurrent increased dramatically at the punch-through voltage (V_p_) of about 20 V; in cases where the reverse voltage was higher than V_p_, the photocurrent was extremely weak. However, as the device was front-illuminated by the full light, photocurrent is clearly present when the reverse voltage is higher than V_p_ of 20 V. In contrast, a similar phenomenon was observed whether the SAGCM InGaAs/InP APDs were front-illuminated or back-illuminated.

**Figure 41:**
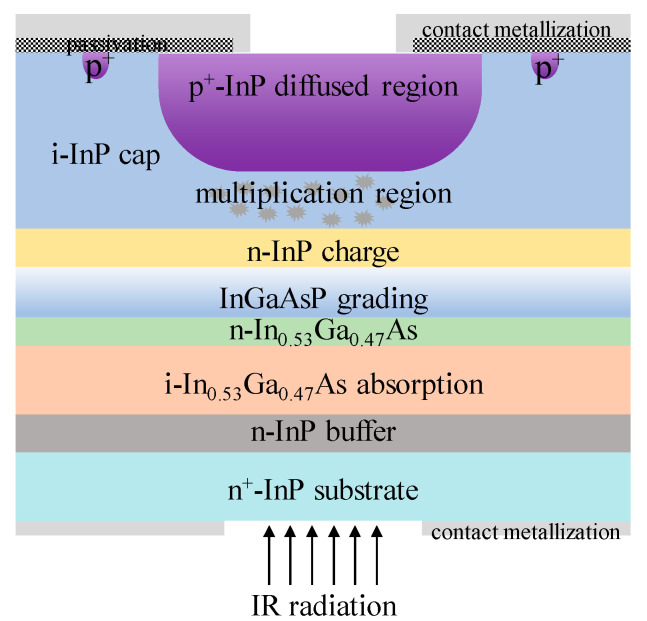
Schematic diagram and SEM image for the optimized back-illuminated planar InGaAs/InP APD.

Then, Yiren Chen et al. [94] proposed the selective-area p-type diffusion method to form the back-side illuminated device, which is clearly observed in Figure 41. Single RTD in N_2_ atmosphere using a Zn_3_P_2_/Zn/SiO_2_ multilayer structure is adopted to realize p-type doping in the InP cap. Extremely high responsivity of 455.5 A/W had been achieved at the reverse voltage of 36.6 V before avalanche breakdown, suggesting that selective-area p-type diffusion is an efficient method to improve the performance of back-illuminated planar InGaAs/InP APD.

**Figure 42:**
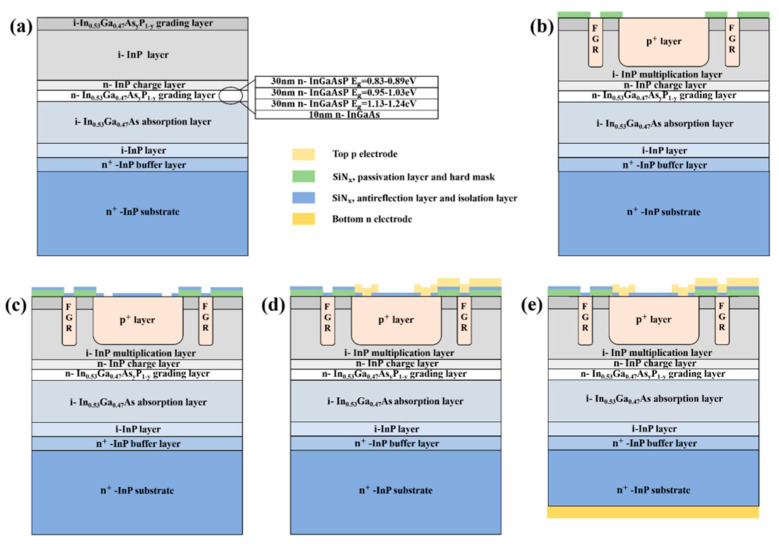
(**a**) Cross-sectional diagram, and (**b**) process flow for the planar geometry SAGCM InGaAs/InP APD with FGR structure, the optimum values for L1 and L2 are 12 μm and 8 μm, respectively(**c**–**e**). Reproduced from [95], IEEE, open access, 2022.

**Figure 43:**
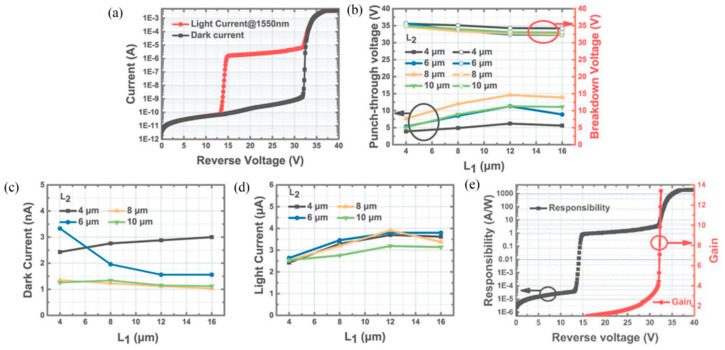
(**a**) I–V characteristics, (**b**) punch-through voltage (V_pt_) and breakdown voltage (V_br_), (**c**) dark current curves, (**d**) light current curves, (**e**) responsivity/multiplication gain vs. reverse voltage. Reproduced from [95], IEEE, open access, 2022.

Junyang Zhang et al. [95] demonstrated the experimental results for the planar InGaAs/InP-based APDs with different FGR structures. Firstly, the MOCVD system was used to grow the SAGCM InGaAs/InP structure on the 2-inch n+-InP substrate (Figure 42a), which is also passivated by the SiN_x_ and it also has the FGR structure (Figure 42b). After the device is fabricated, the diameter of the FGR region and distance between the diffused p+ region and the FGR region are represented as the L1 and L2, respectively. After careful design, the optimum values for L1 and L2 are 12 μm and 8 μm, respectively (Figure 42c–e). Figure 43a shows the typical I-V characteristics under dark and light illumination for the optimized planar InGaAs/InP-based APDs; both V_pt_ and V_br_ can be extracted. The relationship between the Vbr and L1, L2 is plotted in Figure 43b, and it is clearly observed that the V_br_ is less dependent on the values of L1 and L2. The relationship between V_pt_ and L1 and L2 is also observed in Figure 43b, suggesting that V_pt_ varies with L1 and L2. To some extent, the variations of L1 and L2 are helpful for edge breakdown suppression. From Figure 43c, dark currents for the device with the L2 of 8–10 μm remain almost the same, which are slightly higher than 1 nA; light currents for the device with various L1 and L2 are shown in Figure 43d, indicating there is a trade-off value for L1 and L2 towards high-performance devices. Responsivity and efficiency were evaluated to be 9.01 A/W and 72% (multiplication gain equals 10), respectively (Figure 43e).

**Figure 44:**
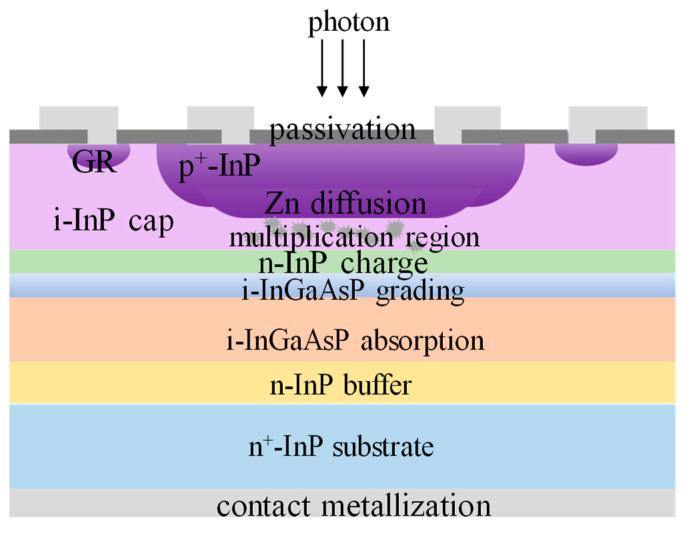
Schematic diagram of front illuminated InGaAs/InP SAGCM SPADs.

F. Signorelli et al. [96] present an InGaAs/InP SAGCM SPAD with high SPDEs and low noise (Figure 44). Improved SPDE is primarily due to the thicker (2 µm) InGaAs absorption region. To avoid the edge breakdown and decrease the charge persistence, a guard ring structure was also introduced. Under the 1550 nm laser illumination, the low temperature (225 K) breakdown voltage and punch through voltage are 68 V and 53 V, respectively. After optimizing the guard ring structure, this device exhibits SPDE up to 53% with a few kpcs DCR at 225 K. The timing jitter is narrow, at 70 ps (FWHM).

#### 2.3.4. Other Novel InGaAs Avalanche Device Structures

**Figure 45:**
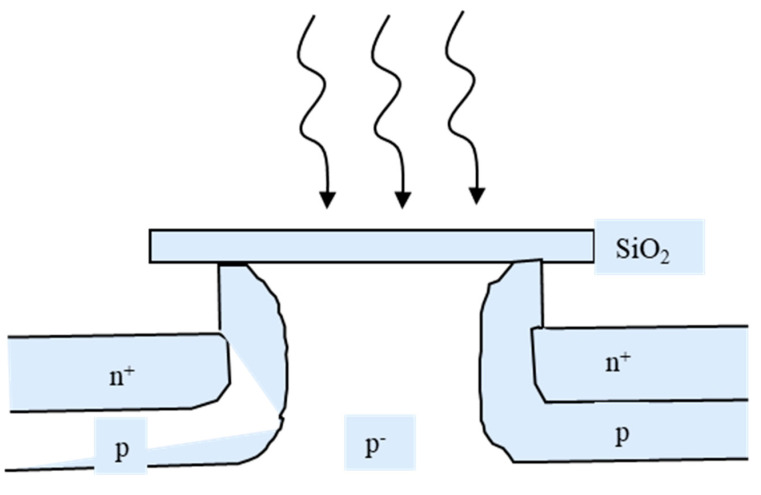
Schematic device structure of Si N+-pp-p-N+ avalanche phototransistors.

The avalanche phototransistors were used as generators of high-current pulses in the early 1970s. As introduced in Ref. [97], the bias voltages applied to the transistors are set slightly lower than the breakdown voltage at that moment. Then, once a beam of light is illuminated on the device, pairs of electrons and holes are generated and separated by the almost breakdown electric field. Therefore, an avalanche process occurs, and a strong current pulse is obtained. The mechanism of such devices is similar to that of Geiger-mode avalanche photodiodes (GM-APDs). In 1981, on the interdigital electrode architecture, the first base-floating N+-pp-p-N+ avalanche phototransistors (APTs) were proposed by Chen, C. W. on Si platform [98] (Figure 45). The design was accomplished with a symmetric N+-pp-p-N+ structure on a (001) p-type Si substrate. The P- and N-type regions are respectively implanted by boron and arsenic atoms, followed by 1100 °C annealing for 4.5 h.

When the device is in operation, the emitter-base junction is forward biased while the opposite p-pn+ junction is reverse biased. To make it work at avalanche mode, the applied voltage should be high enough. When an incident light illuminates the surface of the santireflection layer (SiO_2_), electron-hole pairs are generated. The generated holes are swept and accumulate in the p region, helping the injection of electrons toward the collector. Then, the electrons are swept into the multiplication layer, and an avalanche occurs. In addition, the p-region on the multiplication side is of course necessary to provide a high-strength field when the forward-bias side prevents the punch-through effect from the avalanche field.

**Figure 46:**
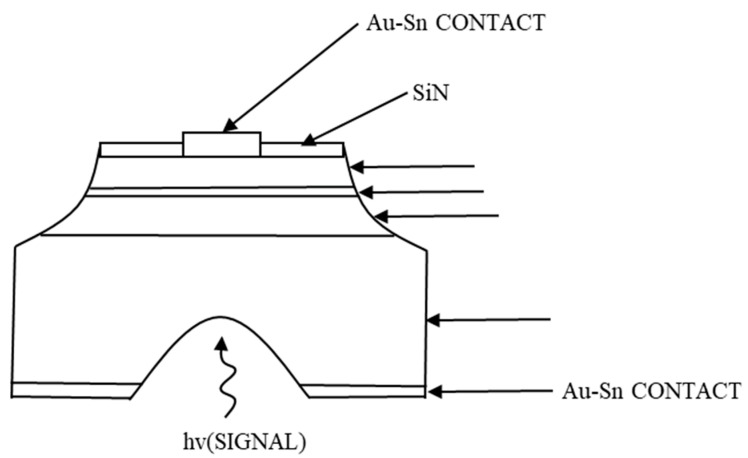
Cross-section schematic diagram of vertical avalanche heterostructure phototransistors.

From 1981, after the work of Chen, C. W., Joe C. Compbell moved the APT design toward the InGaAs/InP material system [99,100]. A vertical NPN structure was utilized in those series papers, instead of the interdigital structure. That is, from the bottom, mainly composed by an n-type InP emitter with donor concentration of 2 × 10^17^~8 × 10^18^ cm^−3^, a thin (~1 μm) p-type In_0.53_Ga_0.47_As base with acceptor concentration of 5 × 10^16^~5 × 10^17^ cm^−3^, and an unintentionally doped (UID) In_0.53_Ga_0.47_As collector with donor concentration of 1~5 × 10^15^ cm^−3^. Figure 46 demonstrates the device cross-section schematic diagram. In the multiplication layer, the secondary holes are swept back to the base. This motion enhances the accumulation of holes inside the p-type region, increasing the injection of electrons from the emitter. The enhanced injection further improves the primary number of electrons before multiplication, which is positive feedback for the device operation mechanism.

**Figure 47:**
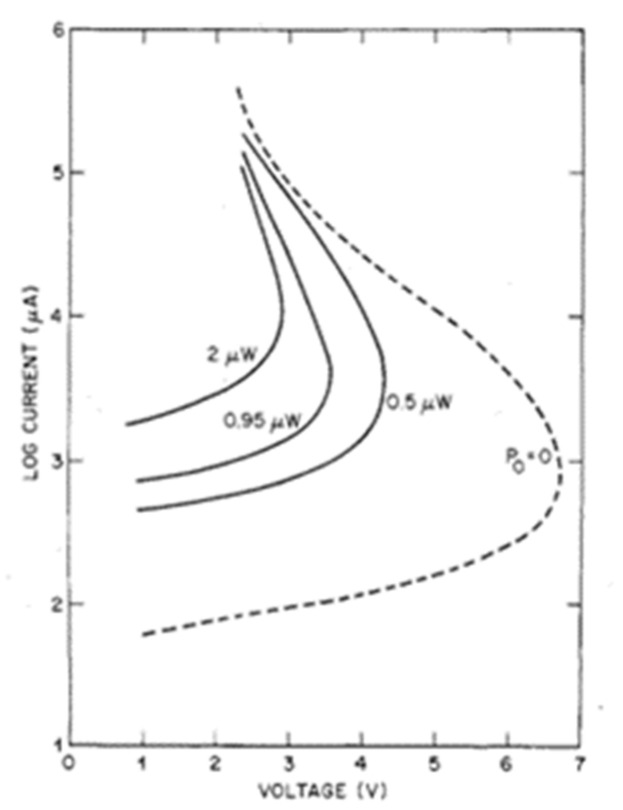
I–V characteristic curves of InGaAs/InP APTs under 0.5 μW, 0.95 μW, 2 μW illumination. Reproduced with permission from [100], IEEE, 2014.

The effective current gain G of an APT was also discussed in the article and given by:

$$G=M\left(h_{FE}+1\right)/\left[1-\left(M-1\right)h_{FE}\right]$$

where *h_FE_* is the common transistor dc current gain and the *M* represents the avalanche multiplication factor. When the term (*M* − 1) *h_FE_* = 1, the current gain, tending to infinity, leads to device breakdown, and the collector current is only limited by series resistance. The corresponding turn-over voltage is denoted by V_t_. When the turn-over voltage excess V_t_ with (*M* − 1) *h_FE_* >1, the dynamic resistance becomes negative, as shown in Figure 47. Under illumination of P_0_ (dark environment), 0.5 μW, 0.95 μW, 2 μW, the current increases with larger applied voltages, and the differential conductivity becomes infinity when bias reaches V_t_. The minus-resistance portions in Figure 47 are obtained by changing the load line, which hints at the decrease of voltage applied to the transistors with higher I_c_.

**Figure 48:**
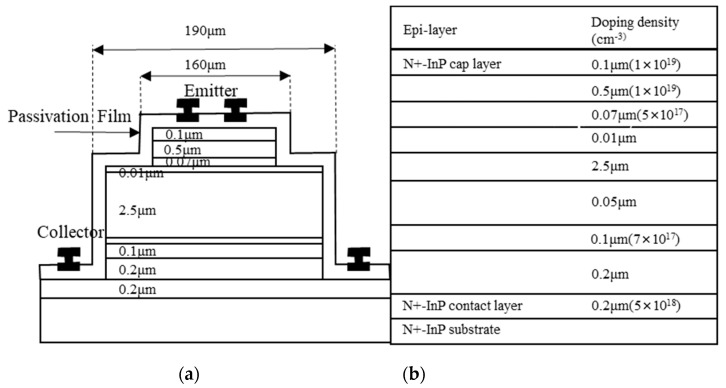
(**a**) The cross-section schematic diagram of APTs and structure profiles, (**b**) bandwidth and response time curves as a function of gain.

Shi, JW improved the structure of III–V APTs by adding In_0.52_Al_0.48_As avalanche p-i-n junction beneath the collector. By this design, they moved the separated-absorption-charge-multiplication (SACM) structure in conventional APDs to the APTs [101]. The schematic cross-section picture and corresponding profiles in each layer are demonstrated in Figure 48a. Upper to the In_0.52_Al_0.48_As avalanche junction, an UID 50-nm graded InAlGaAs layer is inserted to smooth the energy band offset between the In_0.53_Ga_0.47_As collector and the lower In_0.52_Al_0.48_As multiplication layers. The p-type In_0.52_Al_0.48_As charger does not only provide a strong field, but it also prevents the punch-through effect on the intrinsic In_0.53_Ga_0.47_As collector. Figure 48b shows the operation bandwidth as a function of device current gain, concluding that at bias = 6 V, the highest gain-bandwidth couple can be achieved at ~90 THz. A very short response time of about ~160 ps can be obtained with a light pulse of 0.08 pJ/pulse.

In conclusion, for APTs, the gain changes with various incident powers, even at the same bias. This feature is due to the enhanced hole accumulation in the base under illumination, resulting in a higher transistor current gain hFE and a lower sufficient multiplication factor M, e.g., a lower VCE. It means that if APTs are utilized in some applications with uncertain incident power, the applied bias is hard to choose, which would limit the range of applications. In energy-band design, a wider energy-gap material is always utilized as the emitter. Such materials increase the energy barrier between the emitter and base, helping the accumulation of carriers inside the base. In addition, because the avalanche region is induced, the multiplication layer designs of APDs can be taken into consideration, such as the SACM structure, low-k, and wide energy-band materials.

## 3. SWIR APDs Focal Plane Arrays (FPAs)

For sensitivity in the SWIR wavelength range, InGaAs/InP pixel array chips were generally accepted owing to their excellent absorption properties in this range. However, they are not able to be integrated into CMOS. Ge/Si APDs are cheaper to produce and compatible with the traditional CMOS technology, but there are some problems with sensitivity and noise levels in this range. For high-performance Ge/Si APDs, a 4% lattice mismatch between Si and Ge makes the implementation of low-cost pixel arrays more challenging.

### 3.1. Ge/Si APDs FPAs

**Figure 49:**
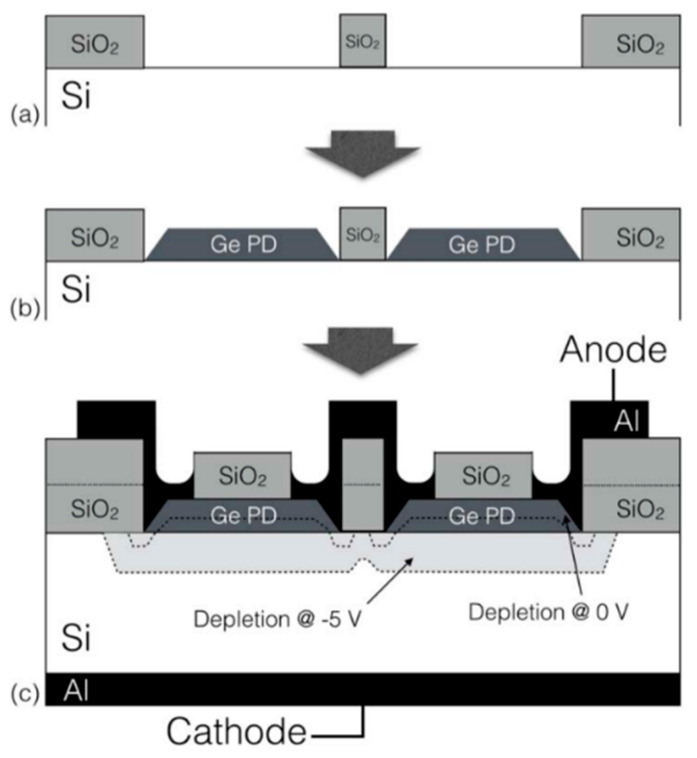
Process flow for PureGaB Ge/Si APDs, (**a**) open the window through SiO_2_ to Si, (**b**) deposit the Ge/Si PDs in the windows, (**c**) form the Al contact. Reproduced from [102], IEEE, open access, 2015.

In 2016, Amir Sammak et al. [102] demonstrated the CMOS-compatible Ge/Si APD pixel arrays. To achieve high-quality Ge/Si APD, the pure gallium and pure boron (PureGaB) Ge/Si process was introduced. This way, the defect level for the Si/Ge interface maintains a very low level after the PureGaB deposition. Arrays of 300 × 1 pixels were integrated on the n-type Si (100) substrate. Figure 49 shows the basic process flow for PureGaB Ge/Si APDs, which is as follows: (I) grow 30 nm thermal SiO_2_ on a Si substrate; (II) deposit 1 μm SiO_2_ on thermal SiO_2_/Si via low pressure CVD; (III) pattern the SiO_2_ layer; (IV) etch on the Si surface and define the APD areas. For the accuracy of the characterization results, it is essential that all the Ge/Si APD pixel arrays were uniformly exposed to the incident light.

**Figure 50:**
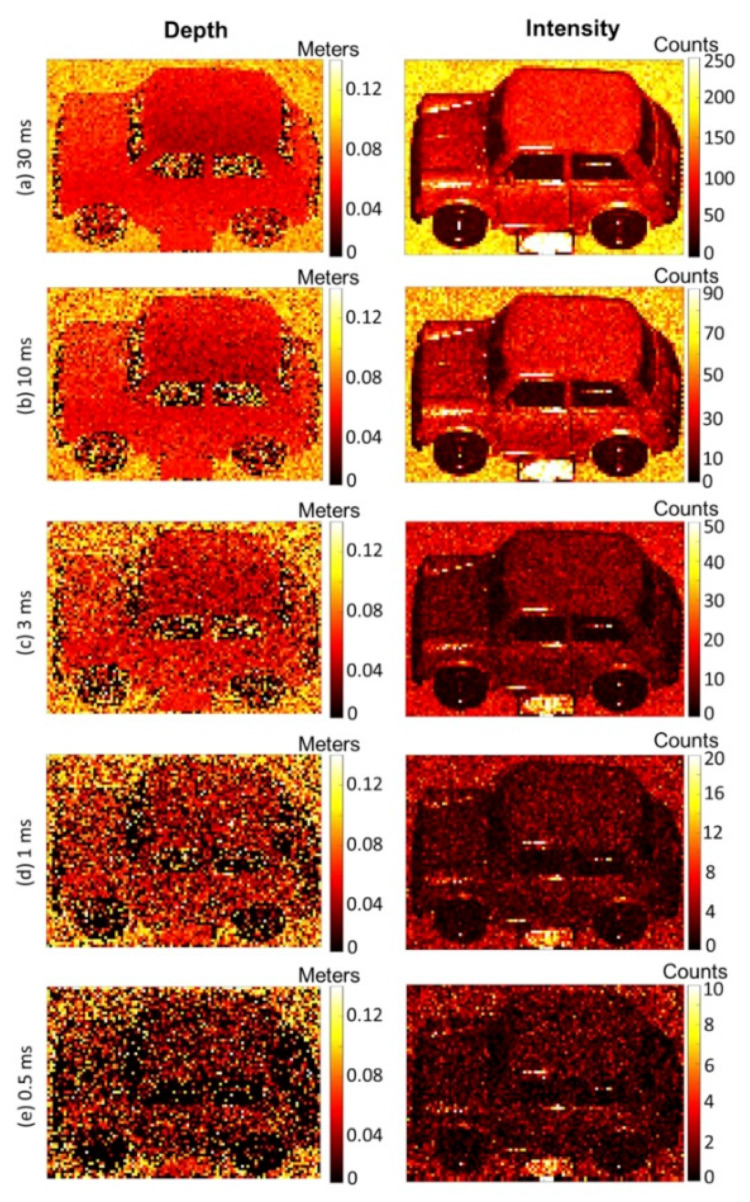
Depth and intensity profile measurements were reconstructed using the pixel-wise cross-correlation approach with the per-pixel acquisition times. Reproduced from [103], Optica Publishing, open access, 2020.

Based on the above-mentioned planar Ge/Si SPAD structure, detector arrays can be fully processed by Si CMOS technology. In 2020, KATERYNA et al. [103] reported the demonstration of LIDAR 3D imaging using planar geometry Ge/Si SPAD at a wavelength of 1450 nm (“eye-safe” consideration). Figure 50 shows the depth and intensity profile measurements reconstructed using the pixel-wise cross-correlation approach by altering the per-pixel acquisition time. For a pixel acquisition time of 0.5 ms, the average number of photons per pixel over the entire scene was 1.4 photons per pixel. As expected, the quality of image reconstruction degrades as the acquisition time is reduced, becoming difficult to discern in isolation at the 0.5 ms acquisition time per pixel. Several algorithms have been developed to restore depth and intensity images in extreme cases, such as photon-starved regimes, including RDI-TV, ManiPoP, UA, and NR3D. In this paper, we highlight the benefit that both the RDI-TV algorithm and the ManiPoP algorithm provide in reducing the acquisition time.

### 3.2. InGaAs APDs FPAs

Since InGaAs/InP and InGaAs/InAlAs SAGCM APDs have been studied for several decades, which means there are already mature products on the market [104,105,106]. Herein, typical InGaAs APD FPAs were selected to review its current progress.

**Figure 51:**
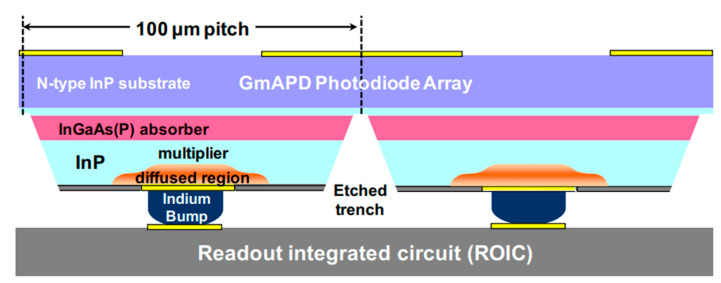
Schematic diagram of planar-geometry InGaAs/InP APD FPAs contacted to CMOS readout ICs via in-bump flip-chip bonding. Reproduced with permission from [107], IEEE, 2010.

**Figure 52:**
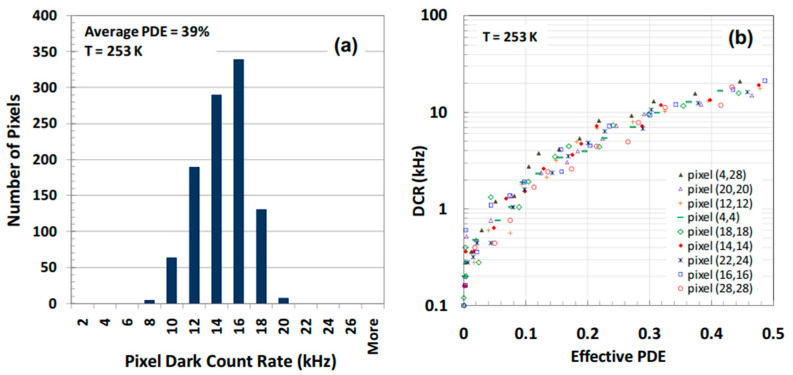
(**a**) Pixel DCRs distribution for the InGaAs/InP FPAs at 1.06 µm; (**b**) SPDE-dependent DCRs. Reproduced with permission from [107], IEEE, 2010.

Mark A. Itzler et al. [107] demonstrated the planar-geometry InGaAs/InP APDs with 32 × 32 FPAs, which are also hybridized to the CMOS ROICs in order to enable the ToF measurement for each pixel (Figure 51). To check the performance, both SPDE measurements and DCR measurements were carried out. Both full FPA maps operating at 1.06 µm and 1.55 µm were demonstrated. For the 1.06 µm FPAs, SPDE is higher than 40% with DCRs of 20 kHz at 250 K (Figure 52a). In the whole FPAs, average DCRs were evaluated to be 13.6 kHz, and a standard deviation of 4.2 kHz also existed (Figure 52b). As for the SPDEs distribution, the average SPDE is around 39%, which has a 6.3 % deviation. Both the deviations of the DCRs and SPDEs were mainly attributed to the variation of the APD breakdown voltage (V_b_), which can be corrected by using non-uniformity correction factors.

**Figure 53:**
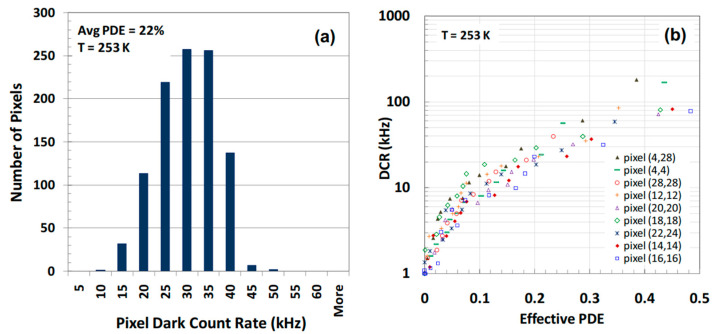
(**a**) Pixel DCRs distribution for the InGaAs/InP FPAs at 1.55 µm; (**b**) SPDE-dependent DCRs. Reproduced with permission from [107], IEEE, 2010.

To meet the eye-safety requirement, 1.55 µm FPAs had also been checked at 250 K. In the whole FPAs, average DCRs were evaluated to be 28 kHz and standard deviation as about 6.5 kHz (Figure 53a). As for the SPDE distribution, the mean SPDE is around 22.2%, which has a 4.6% deviation (Figure 53b). Both the deviations of the DCRs and SPDEs were mainly attributed to the variation of the APD breakdown voltage (V_b_).

### 3.3. Challenges

#### 3.3.1. Challenges for Ge(GeSn)/Si APDs

From all the above-mentioned reported results, Ge/Si APDs are all directly sitting on the Si platform. However, device performance is not available for large-scale commercialization. There are several challenges that need to be overcome.

**Figure 54:**
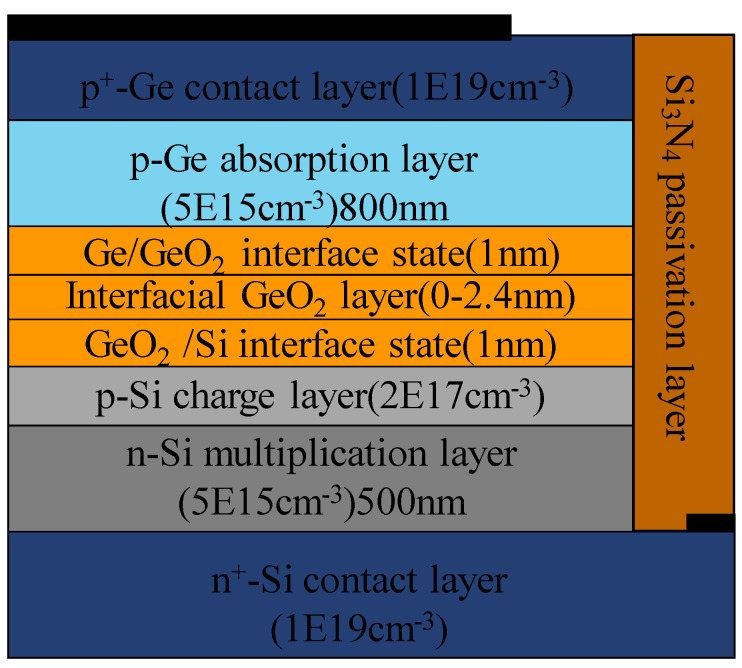
Schematic of wafer-bonded Ge/Si APDs with Ge/GeO_2_ interface state and GeO_2_/Si interface state.

**Figure 55:**
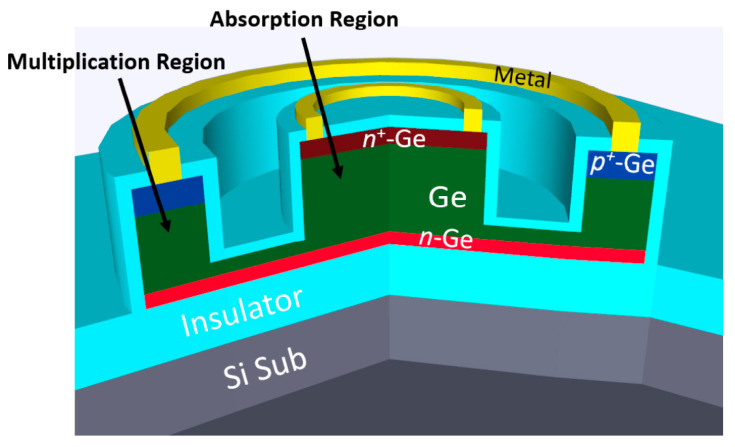
3D Schematic of wafer-bonded Ge APDs on the GOI platform. Reproduced from [125], open access by NTU Library, 2021.

Firstly, TDDs in the Ge absorption layer are still high. To eliminate the TDDs, numerous strategies were proposed in recent decades, including doped Ge buffer layers [108,109,110], compositionally graded SiGe buffer layers [111,112,113], ultra-thin Si/SiGe superlattice buffer layers [114,115], high temperature annealing [116,117,118], three-step growth [119,120], the selective epitaxial growth (SEG) method [121,122,123], etc. With the continuous effort focused on decreasing the TDDs in the Ge layer, the TDDs for the top Ge layer were evaluated to be in the orders of 10^6^–10^7^ cm^−2^. However, the defective level between the Si substrate and Ge layer is extremely high (usually 10^9^–10^10^ cm^−2^), which hinders the low dark current and low DCR Ge/Si APDs. Therefore, suppression of the dark current density is the main topic for high-performance Ge/Si APDs. To solve this problem, researchers proposed the wafer-bonding technique to achieve the low TDDs of the Ge layer on the insulator, which is expected to decrease the dark current as much as possible. Based on the wafer-bonding technique, Shaoying Ke et al. [124] theoretically investigated the wafer-bonded Ge/Si APDs in terms of tunneling effect and interface state (Figure 54). A thin GeO_2_ insulator layer is inserted between the Ge absorption layer and the Si charge layer. With the increasing GeO_2_ thickness, photocurrent is greatly decreasing due to the reduction of tunneling possibilities and carrier accumulation in the Ge absorption layer. Furthermore, interface state densities (ISDs) play a vital role in improving the APD gain and 3dB-BW (bandwidth) at lower bias. There is also a novel APD structure proposed on the germanium on insulator (GOI) platform [125], which was shown in Figure 55. However, this structure has not been experimentally achieved yet. More efforts should be made to process the wafer-bonding Ge/Si APDs, which are very important for the commercial application.

**Figure 56:**
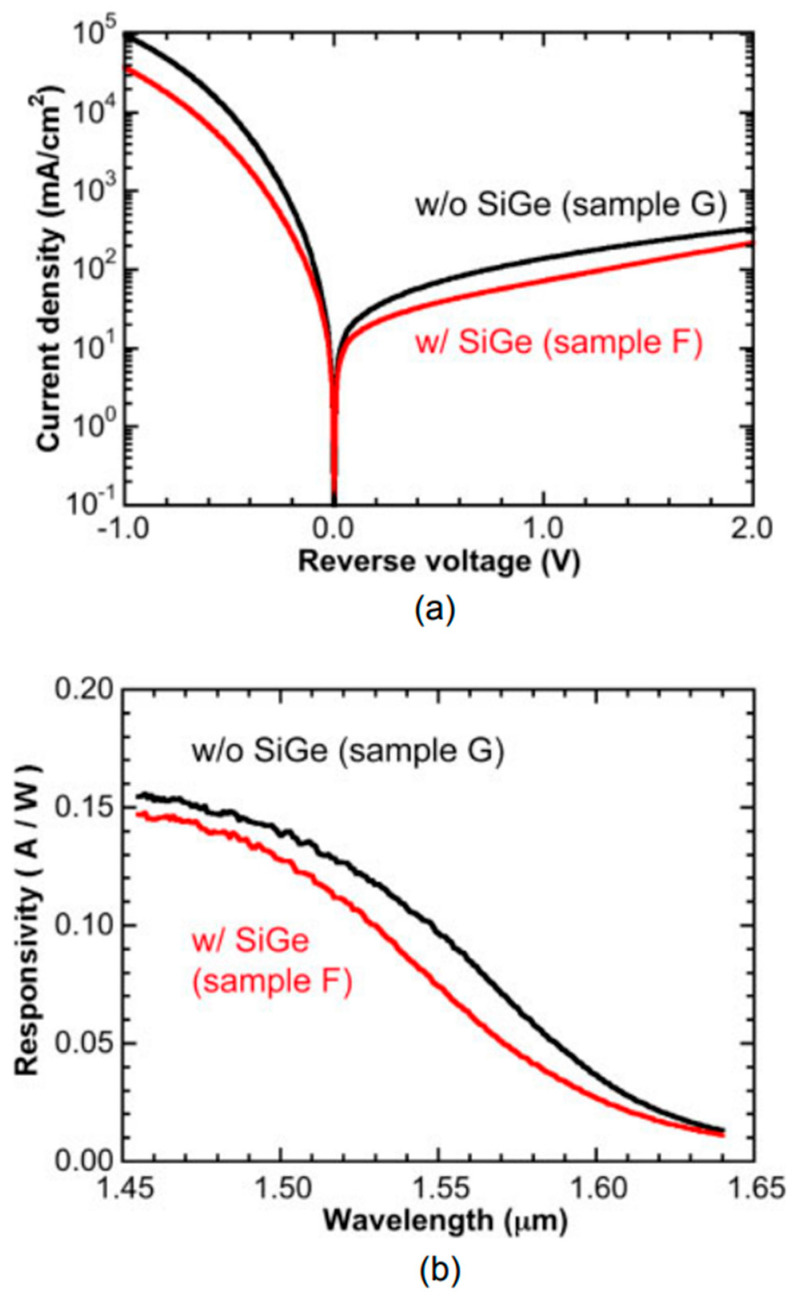
(**a**) Typical I-V characteristics without illumination; (**b**) responsivity spectra biased at 3 V (w/o SiGe and w/SiGe indicate without SiGe and with SiGe, respectively). Reproduced with permission from [126], IOP Publishing, 2016.

Another strategy for optimizing the device performance is the introduction of Ge/graded-SiGe heterostructures in the multiplication layer, which is helpful for controlling the impact ionization. It should be noted that Si is one of the best materials for the multiplication layer in terms of low-noise operation due to its ratio of electron and hole ionization coefficients being far from unity (~0.01). However, smaller ionization coefficients lead to high operation voltages, which are 2~3 times higher than those for Ge/Si APDs. In Ge, the ionization coefficient for holes is slightly higher than that for electrons. Thus, excess noises were reduced by enhancing the ionization coefficient for holes. Using the Ge/graded-SiGe heterostructure in multiplication is one option to suppress the excess noise. Figure 56 shows the typical I–V curves and responsivity spectra biased at 3 V, it can be clearly observed that the performance of each device is comparable, suggesting this strategy is promising for low-noise and low-voltage APD applications [126].

Bandgap properties for GeSn material can be tuned by changing the incorporation of Sn into Ge, which makes it suitable as the absorption layer for the Si-based low-cost SWIR APDs, especially for the eye-safe 1550 nm light detection. To grow the high-quality GeSn materials, several growth methods were explored, including MBE, PVD, and CVD. Up until now, great breakthroughs had been made for high-Sn-composition material growth. Especially for CVD growth, this is the most promising method to grow high-quality materials with high Sn composition [127,128,129,130,131,132,133,134]. From the device perspective, although linear mode GeSn APDs have been demonstrated experimentally, GeSn growth techniques are facing several bottlenecks, such as Sn segregation during growth and after annealing, which leads to severe surface roughness and affects the material properties [135,136,137,138,139,140,141]. Other issues include uniformity in the GeSn absorption layer, Si/Ge interface and Ge/GeSn interface quality, the existence of point defects in direct bandgap GeSn, etc. Similar to the Ge defect reduction strategies, such as doped buffer layers, compositionally graded buffer layers, ultra-thin superlattice buffer layers, high temperature H_2_ annealing, cyclic thermal annealing, and the selective epitaxial growth (SEG) method, were also needed to investigate the defect suppression mechanism for GeSn materials [142]. Apart from the GeSn growth difficulties, there is little theoretical modeling research on the GeSn APDs, which hinders device design and performance prediction. Due to the missing parameter for GeSn, the majority of simulation results are not described accurately [143,144,145,146,147,148]. Therefore, in-depth and accurate GeSn material parameters studies are required, whether experimentally or theoretically.

From the device structure perspective, a large amount of Ge(GeSn) APDs were SACM structures. In this structure, Si was always adopted as the multiplication layer owing to its ionization rate ratio, which varies considerably with electric field. However, the lattice parameter mismatch between Si and Ge(GeSn) is also large. To achieve high performance Ge(GeSn) APDs, it usually needs to weigh the advantages and disadvantages of the Si multiplication layer and defect level among the total device structure (Si/Ge interface and Ge absorption). To give attention to these two factors, more innovative devices are required to be proposed. Moreover, only a few groups are working on the planar-geometry Ge(GeSn) APDs, but more efforts should be devoted due to its excellent SPDE.

Emerging group IV material systems, such as GeSnSi alloys [149], GeSnSiC alloys [150,151], GePb alloys [152,153,154], and GeSnPb alloys [155], are also promising SWIR absorbers towards high-performance APDs. Especially for the GeSnSiC alloys, Ge_1−x−y−z_Sn_x_Si_y_C_z_ films (0.01 ≤ x ≤ 0.06, 0 ≤ y ≤ 0.02 and 0 ≤ z ≤ 0.01) had been successfully grown at 280–330 °C on Ge and Si using commercial RPCVD technique, indicating it has the great potential to be commercialized. Moreover, GeSnSiC alloys are also important for future Si-based monolithic integration [156]. To further improve the device performance, quantum well structures [157,158] and quantum dots multilayer structures [159] are also feasible for the enhancement of SWIR detection. For the APD devices, the multiplication effect also plays a vital role in the device’s performance. However, the large lattice mismatch between Si and Ge(GeSn) is the main hurdle for the high-performance Ge(GeSn) APDs. To balance the multiplication effect and lattice mismatch problem, the SiGe layer is also an important candidate for the multiplication region due to its CMOS process compatibility, relatively strong avalanche effect, and smaller lattice mismatch with Ge and GeSn [160,161,162,163]. Moreover, SiGeC is a conspicuous choice for the multiplication region, which is helpful for the temperature coefficient of resistance and low noise level [164,165]. To lower the contact resistance, the nickel strategy is widely used at low temperatures (compared to Co) for forming the Ni-Si, Ni-SiGe, Ni-Ge, and Ni-GeSn ohmic contacts [166,167,168,169,170,171].

#### 3.3.2. Challenges for InGaAs/InP APDs and InGaAs/InAlAs APDs

From the reviewed articles above, it was clear that the majority of the InGaAs APDs were grown on the InP substrate by the MBE and MOCVD tools. As is known to all, InP substrates are smaller and extremely expensive, which makes their commercial cost very expensive. To push the InGaAs APDs towards cost-effective LiDAR applications, several problems need to be solved.

Firstly, low-cost and large size wafer should be developed, such as, GaAs and Si. For the GaAs case, wafer size can reach up to 6 or 8 inches now, but there is lattice mismatch and thermal mismatch between the GaAs substrate and InP layers; for the Si case, there are always 8 inch, 12 inch, or even 18 inch wafers, which also have the problems of thermal mismatch, lattice mismatch, and polarity contrast between the Si substrate and InP layers. Compared to the GaAs substrate, there are more problems that need to be dealt with for the Si substrate. More explorative investigations should be conducted to overcome the growth obstacles. Thus, cost-effective InGaAs APDs will be achievable, and the LiDAR price will also be lower [172,173,174,175,176,177,178,179].

**Figure 57:**
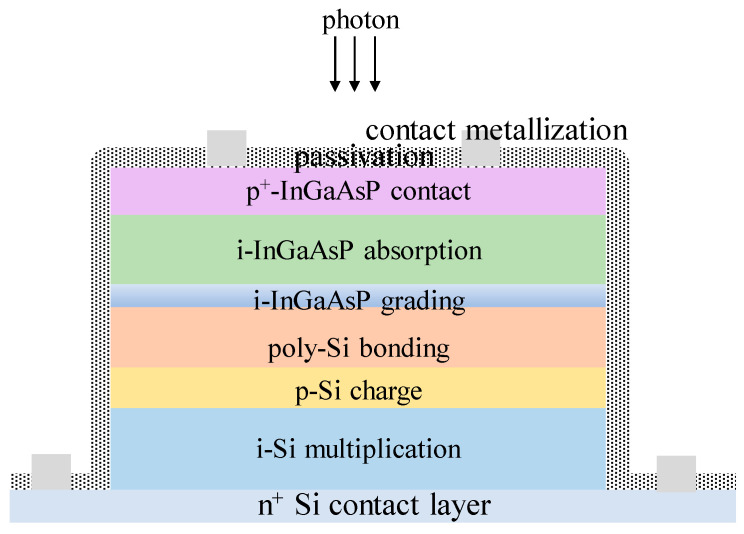
Wafer-bonded InGaAs/Si SAGCM APDs.

Secondly, InGaAs/InAlAs APDs have the great potential to substitute the InGaAs/InP APDs, which makes it very important to grow the high-quality InAlAs multiplication layers. Although InGaAs/InP APDs have been commercialized for a long time, there is still room to improve the device’s performance. Especially from the defect reduction and multiplication gain enhancement perspectives, which will greatly improve the device performance. Until now, InP and InAlAs layers were employed as the multiplication region; Si is also a promising candidate for the multiplication region [180,181,182,183,184,185]. Wafer-bonded InGaAs/Si APDs were proposed to greatly improve the device performance (Figure 57), which gives researchers a novel technical route to optimize the device architecture [186].

Despite the growth and device structure issues, effective passivation technology is of great significance to improving the performance and stability of the device. The properties of many semiconductor devices are closely related to the surface properties of semiconductors. In some cases, it is often not the in vivo effect of semiconductors but the surface effects that govern the characteristics of semiconductor devices [187]. For mesa-type infrared detectors, surface properties are often the key factor affecting the final performance of the device. The quality of the surface condition is an important source of device noise, and it is an important factor causing the crosstalk of multiple components. Large-scale FPA detectors with smaller spacing can now be created, thereby improving the resolution of the image. However, as the spacing of the mesa detector arrays decreases, the signal-to-noise ratio also decreases [188]. The mesa detector etching process resulted in the appearance of dangling bonds on the side walls, which causes a high surface leakage current [189]. These bonds hanging from the surface of the InGaAs region of the InGaAs/InP photodetector significantly affect the stability of the dark current [190]. Surface leakage has a great impact on the performance of photovoltaic devices. At the same time, the size of the surface composite speed determines the performance of the optical conductivity device. The high surface density of states and the surface composite rate have been restricted to the development of III–V family semiconductor devices, and they need to be solved by surface passivation. The purpose of passivation is to protect the surface from external pollution and damage and reduce the surface density of states, surface recombination rate, and side leakage current, so as to reduce the dark current of the detector [191], improve the detection rate, and ensure the long-term stability of the device. In order to obtain excellent semiconductor surface interface properties, on the one hand, it is necessary to effectively remove the semiconductor surface pollutants to achieve a pure surface and effectively prevent a pure semiconductor surface from being exposed to air or insulation layer interface pollution or oxidation, namely chemical passivation; on the other hand, it is necessary to effectively reduce the interface state density between the insulating layer and the semiconductor material, namely electrical passivation. The passivation process is an essential and very critical process for semiconductor device preparation, and tremendous studies have been conducted and many effective methods have been summarized.

Before growing passivation films using techniques such as chemical vapor deposition, atomic layer deposition, or organic passivation and surface thiolation, it is often necessary to perform some auxiliary treatment on the semiconductor surface first to remove surface pollutants, impurity ions, and the air oxide layer. The common methods mainly include chemical solvent treatment, plasma cleaning, special gas treatment, and light treatment [192,193,194,195,196,197,198]. (a) Chemical solvent treatment, chemical solvents treatment commonly used organic solvents are: alcohol, acetone, methanol, trichloroethylene, etc.; inorganic solvents include dilute hydrochloric acid, sodium hydroxide/potassium, hydrofluoric acid, and other solutions. These solvents can effectively remove the vast majority of organic matter, oxides, and impurities on the semiconductor surface. (b) Plasma cleaning, of the adhesion force of the passivation layer film to the semiconductor surface, is not only dependent on the type of substrate material but is also affected by the surface residues. Before depositing the passivation film, the plasma precleaning of the material surface can effectively improve the adhesion force between the film and the substrate material. Studies have shown that hydrogen plasma can effectively remove inorganic residues, and oxygen plasma can effectively remove organic residues. The hydrogen plasma pre-cleaning process before the film deposition can effectively improve the quality of the deposited film and its adhesion to the substrate material. For example, the NH_3_ plasma pre-cleaning process can decompose NH_3_ into N and H atoms. H atoms acting on the surface can form a hydrogenated surface layer, forming a beneficial interlayer between the film and the substrate. Jianqiang Lin et al. studied PH_3_ plasma passivation on the InGaAs surface of the MOS device. (c) Special gas treatment is similar to plasma cleaning, before depositing the film, the semiconductor surface through H_2_; trimethyl aluminium (TMA) can also play a certain role in removing oxides, impurities, and dangling bonds. Wipakom Jevasuwan et al. studied the TMA passivation process and interface formation process of an InGaAs MISFET device. (d) Light treatment: R.Driad et al. studied the passivation process of the material surface of InGaAs in the InGaAs/InP structure HBT device and used ultraviolet radiation (UV) and ozone (O_3_) treatment to effectively remove the organic and inorganic materials on the semiconductor surface. Through XPS analysis, it was found that the UV-O_3_ treatment removed the surface defect layer, forming oxides such as As_2_O_3_, As_2_O_5_, In_2_O_3_, and Ga_2_O_3_. The semiconductor surface material components basically showed the stoichiometric ratio, and the device performance was also significantly improved. For III–V compound semiconductor devices, the commonly used medium passivation film mainly includes SiN_x_, SiO_2_, A1_2_O_3_, BCB, Polyimide (PI), and other organic passivation films, sometimes using SiO_2_-SiN_x_ or SiO_2_-PI multilayer passivation film structures. The combined use of two passivation films can effectively achieve the advantages of two films while also achieving stress compensation.

Ohmic contact is one of the most important processes in the preparation of semiconductor devices, which has an important influence on the performance, reliability, and stability of the devices. Good metal-semiconductor ohmic contacts can be obtained in the following ways: (a) Low-barrier connection. If the barrier formed by the formation between the metal and semiconductor contact is low, there are enough carriers at room temperature to enter the metal from the semiconductor or from the metal into the semiconductor; such contacts have minimal rectification effect. (b) High composite contact. The introduction of many strong composite centers in the vicinity of the metal-semiconductor contact surface constitutes a high composite contact. The high composite contacts do not inject the nonequilibrium carriers into the semiconductor, because the original nonequilibrium minority carriers that may have been injected into the body are lost when they pass through the high composite contact zone. The high composite contacts will also not have a rectification effect because, in the reverse, the high composite center will become the high generation center, making the reverse current change very large, and the reverse high resistance state will not exist. If the semiconductor surface is damaged, crystal defects may form near the surface, and their role is similar to that of the compound center. If the composite center density is high enough, the recombination in the depletion zone will become the main conductive mechanism, so that the contact resistance decreases significantly. (c) High doping contact. At the metal contact position of the semiconductor surface and the metal, if a high concentration of donor or acceptor impurities is added first, the high doping contact is formed. On the one hand, the highly doped n+ or p+ layer can effectively reduce the injection of nonequilibrium carriers, while the depletion layer region is very thin and easy to produce field emission, so the contact has a very low resistance at zero bias.

The characterization of metal-to-semiconductor contact characteristics is the specific contact resistance per unit area metal-to-semiconductor contact differential resistance. Specific contact resistance, or the interface resistance of ohmic contact, cannot be directly measured. The contact zone generally includes a metal layer, a metal-semiconductor interface, and a semiconductor junction. In addition, various parasitic resistances have been introduced. Currently, many testing methods based on different models are already available. However, no matter what the method, under a certain constant current, measure the voltage between some contact points, find the respective resistance, and then according to different physical models, from the total resistance deduct various parasitic resistances, and finally achieve the specific contact resistance. For family InP compound semiconductors, P-type ohmic contact is generally much more difficult to make than n-type, firstly because the effective mass of the hole is larger than the electron, and secondly because it is determined by the device processing process itself [199,200,201]. We also summarize the evidence and the pros and cons for Ge (GeSn) and InGaAs APDs in Table 3.

## 4. Conclusions and Perspectives

In this article, we review the recent research progress for the mesa- and planar-geometry InGaAs APDs, which are commonly grown by the MBE or MOCVD technique on the InP substrate. Due to the lattice match between InGaAs and InP or InAlAs, both InP or InAlAs and InGaAs were employed as the multiplication region and absorption region, respectively. To improve the device performance, SAGCM structure InGaAs/InP APDs were generally acknowledged. For the InGaAs APDs with the InAlAs multiplication layer, a higher gain at a low dark current level is obtained. Therefore, InP is gradually replaced by the InAlAs multiplication region owing to its superior ionization coefficients, which reduce the excess noise. At the same time, InAlAs offers a wider bandgap, which is also helpful for reducing the tunneling current. InGaAs APDs with both mesa- and planar-geometry were surveyed, including front-side illumination, multi-mesas, MIM optical microcavities, backside illumination, double-side illumination, FGR structure, and APTs. Although InGaAs APDs have been commercialized for a long time, more explorative investigations should be conducted to overcome the growth obstacles, which will greatly knock down the price of InGaAs APDs.

The Ge (GeSn) material for the APD application has also been reviewed. To improve the device performance, several strategies are proposed, including planar-geometry, an enhanced multiplication layer, photo-trapping microstructure, SEG growth, a multi-mesa structure, photonic crystal enhancement, etc. Among all these strategies, planar geometry features the best performance in terms of SPDE, DCRs, NEP, jitter, and 3D imaging quality. However, the main obstacle to the commercialization of Ge(GeSn)/Si APDs is the high TDD in the whole Ge(GeSn) structure, which leads to high DCRs. Until now, the majority of Ge(GeSn)/Si APDs used Si as the multiplication region. Therefore, there is an urgent need to design and develop more innovative GeSn(Sn) APD structures, which would further pave the way for the huge advancement of the next generation of high-sensitivity SWIR detection systems.

## Figures and Tables

**Table 2:** Summarized device performance for planar geometry Ge/Si SPAD at 1.31 μm [77,78,79].

Diameter (μm)	Temperature (K)	SPDE (%)	DCRs	Jitter (ps)	NEP (WHz^−1/2^)
100	125	38	A vast improvement when compared to previous Ge-on-Si work, staying below 100 kcps for an excess bias of up to 6.5%	310	1.9 × 10^−16^@78 K
	100	26			
	80	22			
50	125	29	Approximately 4 times greater than the 26 µm device at each excess bias level recorded (380 kcps)	210 ± 10	1.6 × 10^−16^
26	125	28	DCRs observed from the 26 µm device were extremely low (86 kcps)	157 ± 10	9.8 × 10^−17^

**Table 3:** Summarized evidence of the pros and cons for Ge (GeSn) and InGaAs APDs.

Index	Ge (GeSn) APDs	InGaAs APDs
Growth technology	MBE, RPCVD	MBE, MOCVD
Substrate	Si	InP
Device structure	SACM	SAGCM
Multiplication region	Si	InAlAs or InP
Absorption region	Ge (GeSn)	InGaAs
Wafer size	8–12 inch available	2–4 inch
Price	Low	High
Wavelength range	1.1–3 μm	1–2.5 μm
Technology Maturity	Research and Development	Commercialization
Product	No	Yes

## Data Availability

The data presented in this study are available on request from the corresponding authors.
